# The Impact of Genetic Variations on Radiotherapy Toxicity in Breast Cancer Patients: A Meta-Analysis of Acute and Late Skin Adverse Effects

**DOI:** 10.3390/cancers17111880

**Published:** 2025-06-04

**Authors:** Andreea Cătană, Andrada-Adelaida Pătrășcanu, Daniela Laura Martin, Mariela Sanda Militaru, Irina Ioana Iordănescu, Alexandru Țîpcu, Patriciu Achimaș-Cadariu, Lorin-Manuel Pîrlog

**Affiliations:** 1 Department of Molecular Sciences, Faculty of Medicine, University of Medicine and Pharmacy “Iuliu Hațieganu”, 400012 Cluj-Napoca, Romania; catanaandreea@elearn.umfcluj.ro (A.C.); sanda.militaru@umfcluj.ro (M.S.M.); 2 Department of Oncogenetics, Institute of Oncology “Prof. Dr. I. Chiricuță”, 400015 Cluj-Napoca, Romania; 3 Regional Laboratory Cluj-Napoca, Department of Medical Genetics, Regina Maria Health Network, 400363 Cluj-Napoca, Romania; 4 Department of Radiotherapy, Institute of Oncology “Prof. Dr. I. Chiricuță”, 400015 Cluj-Napoca, Romania; drdanielamartin@yahoo.com (D.L.M.); alextipcu@gmail.com (A.Ț.); 5 Genetic Centre Laboratory, Department of Medical Genetics, Regina Maria Health Network, 011376 Bucharest, Romania; irina.iordanescu@reginamaria.ro; 6 Department of Oncology, Faculty of Medicine, University of Medicine and Pharmacy “Iuliu Hațieganu”, 400012 Cluj-Napoca, Romania; 7 1st Department of Oncologic Surgery, Institute of Oncology “Prof. Dr. I. Chiricuță”, 400015 Cluj-Napoca, Romania; pachimas@umfcluj.ro

**Keywords:** single-nucleotide polymorphisms, DNA repair pathways, inflammation genes, oxidative stress response, circadian rhythm, personalized oncology, radiogenomics, germline biomarkers, treatment-related toxicity

## Abstract

**Background/Objectives:** Radiotherapy is a cornerstone in the treatment of breast cancer, yet its use is frequently accompanied by skin toxicities that vary in severity and timing among patients. The objective of this meta-analysis is to systematically evaluate the pooled impact of genetic variations on the risk and severity of acute and late skin side effects from radiotherapy in breast cancer patients. **Materials and Methods:** A systematic literature search was conducted across PubMed, Embase, and Scopus to identify studies published between 2014 and 2024 that examined associations between genetic polymorphisms and radiotherapy-induced skin toxicity. Studies were selected based on predefined inclusion and exclusion criteria, and data were synthesized using a random-effects meta-analysis model. The risk of bias was evaluated using the ROBINS-I tool, and publication bias was assessed through funnel plots and Egger’s test. **Results:** A total of 11 studies involving breast cancer patients were included, identifying associations between various gene polymorphisms and skin toxicity. The pooled analysis revealed that patients with specific genetic variants had a 53% increased risk of acute skin side effects and a 44% increased risk of late effects. Notable implicated genes included XRCC2, IFNG, ATM, TGFB1, and PER3. Significant heterogeneity and publication bias were noted across studies, warranting cautious interpretation. **Conclusions:** This meta-analysis highlights the role of genetic variation in predicting radiotherapy-induced skin toxicity in breast cancer patients. These findings support the future development of predictive biomarkers and personalized radiotherapy strategies to minimize treatment-related toxicity.

## Simple Summary

Radiotherapy is a key treatment for breast cancer, but it can lead to skin reactions that differ significantly between patients. Some develop redness or irritation during treatment, while others experience long-term effects like scarring or tissue changes. These side effects are not fully explained by treatment dose alone. This study reviews the role of inherited genetic differences in how patients respond to radiotherapy. By analyzing results from multiple clinical studies, we identify specific genes that may increase or reduce the risk of short-term and delayed skin problems after treatment. Understanding these genetic factors could help doctors predict which patients are more likely to experience severe side effects. In the future, this knowledge may allow for more personalized radiotherapy plans that aim to protect healthy tissue while still effectively treating cancer.

## 1. Introduction

Breast cancer remains one of the most prevalent malignancies worldwide, with radiotherapy serving as a cornerstone in the multidisciplinary management of the disease [1]. While advancements in radiotherapy techniques have significantly enhanced tumor control and survival rates, acute and late side effects continue to be a considerable clinical challenge [1,2]. Acute skin toxicities often arise during treatment, whereas late skin toxicities may develop months to years post-therapy [2].

The interpatient variability in the occurrence and severity of these skin side effects suggests that individual genetic predisposition may play a pivotal role [3]. Over the past decade, a growing body of research has investigated the influence of genetic mutations and polymorphisms on adverse effects following radiotherapy in breast cancer patients. Single-nucleotide polymorphisms (SNPs) in genes involved in DNA repair, oxidative stress response, and inflammation pathways have been associated with varying degrees of skin toxicity [4,5,6,7]. However, findings across studies are often inconsistent and sometimes contradictory, highlighting the need for comprehensive synthesis and analysis.

This meta-analysis aims to systematically review and quantitatively assess the pooled impact of genetic variations on the risk and severity of acute and late skin side effects of radiotherapy in breast cancer patients. Understanding these associations holds great clinical importance, as it may guide the development of predictive biomarkers for radiosensitivity, enabling personalized radiotherapy protocols that minimize toxicity while preserving therapeutic efficacy. Moreover, identifying genetic factors influencing skin adverse effects could foster the creation of novel therapeutic interventions to mitigate these effects.

By consolidating evidence from diverse studies, this meta-analysis seeks to provide a clearer understanding of the genetic determinants of skin radiotherapy-induced toxicities in breast cancer patients, ultimately contributing to more individualized and safer treatment approaches.

## 2. Materials and Methods

### 2.1. Protocol and Guideline Compliance

This review was conducted in accordance with the PRISMA 2020 guidelines to ensure a transparent, comprehensive, and methodologically sound synthesis of the available evidence [8]. Although no formal protocol was registered prior to the initiation of the review, all relevant steps were carried out following established best practices to maintain the integrity and reliability of the systematic process.

### 2.2. Literature Search Strategy and Database Selection

A comprehensive literature search was conducted to identify relevant studies exploring the relationship between breast cancer, radiotherapy, radiation side effects, and genetic factors. The search strategy was designed using a logical formula comprising multiple keywords and Boolean operators to ensure the inclusion of all relevant studies.

The search formula employed was as follows: (Breast cancer OR Breast carcinoma OR Breast neoplasm OR mammary neoplasm OR mammary cancer) AND (Radiotherapy OR Radiation therapy OR Radiation treatment) AND (Radiation skin side effects OR Radiation-induced skin toxicity OR Skin adverse effects of radiotherapy) AND (Genetics OR Genetic predisposition OR SNP OR DNA repair OR Genetic markers OR Genomic variation).

The search was performed across three major scientific databases: PubMed, Embase, and Scopus. These databases were selected for their extensive biomedical and clinical research coverage, including peer-reviewed journal articles, conference proceedings, and other scholarly publications.

The search strategy aimed to capture articles discussing the genetic predisposition and genomic variations influencing the risk of developing adverse skin effects due to radiotherapy in breast cancer patients.

### 2.3. Selection Criteria Overview

The selection criteria for this review were established to ensure the inclusion of relevant studies focused on the relationship between genetic factors and radiotherapy-induced toxicity in breast cancer patients. Studies were selected based on the following inclusion and exclusion criteria:

- Inclusion criteria:
  a.1. Studied population: articles involving breast cancer patients who have undergone radiotherapy;
  a.2. Genetic focus: research examining genetic markers or genetic variations associated with skin radiotoxicity;
  a.3. Study design: case-control studies, observational studies, or randomized controlled trials;
  a.4. Outcomes studied: studies investigating the correlation between genetic markers and skin radiotherapy-induced side effects, including both acute and late-onset toxicity;
  a.5. Language: articles published in English;
  a.6. Human studies: studies conducted exclusively on human participants;
  a.7. Publication date range: articles published between 1 January 2014, and 31 December 2024;
- Exclusion criteria:
  b.1. Non-breast cancer populations: studies focused on cancers other than breast cancer;
  b.2. Lack of genetic data: articles lacking information on genetic markers or genetic variations relevant to skin radiotoxicity;
  b.3. Preclinical studies: research involving animal models or in vitro studies;
  b.4. Radiotherapy without genetic focus: studies discussing skin radiotoxicity in breast cancer patients without examining genetic predispositions;
  b.5. Language restrictions: articles published in languages other than English;
  b.6. Ineligible works: meta-analyses, systematic reviews, reviews, case reports, conference abstracts, encyclopedia articles, book chapters, posters, or oral presentations addressing genetic factors influencing radiotoxicity in breast cancer patients;
  b.7. Chemotherapy toxicity: studies investigating toxicity associated with chemotherapy or other systemic therapies (e.g., hormonal or immunotherapy);
  b.8. Out-of-date publications: articles published before 1 January 2014, or after 31 December 2024.

### 2.4. Article Selection Process

To ensure a rigorous and systematic literature review, a comprehensive search was conducted across three major databases: PubMed, Scopus, and Embase. Specific exclusion criteria were used to filter out studies that did not align with the research objectives. This selection process adhered to the PRISMA (Preferred Reporting Items for Systematic Reviews and Meta-Analyses) guidelines, as illustrated in the flowchart.

Initially, 2329 records were retrieved from the selected databases: 880 articles from PubMed, 833 articles from Scopus, and 616 articles from Embase. After 253 duplicate records were removed, 2076 reports were subjected to title and abstract screening.

During this screening phase, reports were excluded for various reasons, including studies focusing on non-breast cancer populations, lacking relevant genetic data, involving preclinical studies, not analyzing genetic predispositions to skin radiotoxicity, language restrictions, ineligible publication types, exclusive focus on chemotherapy toxicity, or falling outside the specified publication date range.

The remaining 24 reports underwent a more detailed full-text analysis to assess their eligibility. Ultimately, 11 studies were deemed suitable for inclusion in the final review, meeting all predefined inclusion criteria.

Two authors, L.-M.P. and A.-A.P., independently carried out all stages of the screening process, including title and abstract screening and full-text assessment, following the predefined inclusion and exclusion criteria. To enhance the accuracy and reliability of the selection process, a third author, A.C., was consulted whenever disagreements arose between the initial reviewers.

In cases of discrepancy, the third author carefully reassessed the study in question, evaluating its eligibility based on the established criteria. The final decision regarding the inclusion or exclusion of the study was made using a majority-rule approach (2 out of 3 agreements). This method ensured that each disputed study was thoroughly examined and fairly assessed, thereby minimizing potential biases, and enhancing the overall rigor of the selection process.

By implementing this collaborative and structured approach, the review process maintained high consistency, transparency, and methodological integrity.

**Figure 1:**
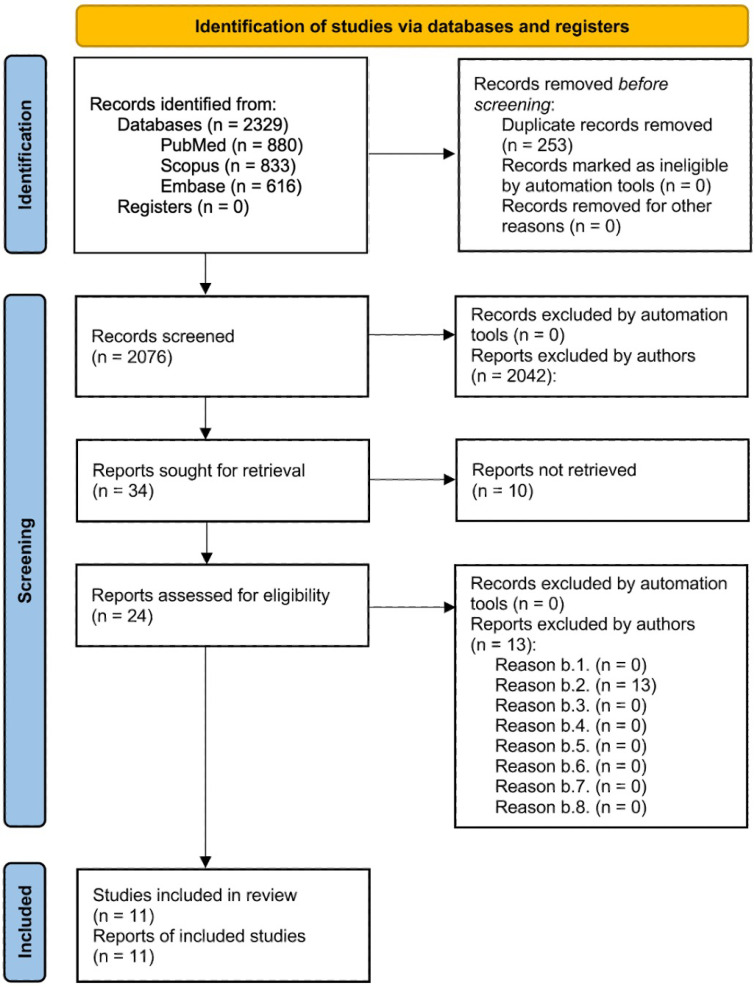
PRISMA flow diagram illustrating the article selection process. This diagram provides a comprehensive overview of the article selection process according to the PRISMA guidelines, detailing the number of records identified, duplicates removed, reports screened, and studies included in the final review. Exclusion reasons are categorized and quantified at each stage to ensure transparency and rigor in the selection process.

The PRISMA flowchart provides a visual summary of the selection process, detailing the number of records retrieved from each database, duplicates removed, and the filtering process through title and abstract screening followed by full-text assessment. The reasons for exclusion are categorized at each stage, enhancing transparency and clarity regarding the selection process. This structured approach ensures that only high-quality, relevant studies contribute to the review, thereby enhancing the reliability and validity of the findings (Figure 1).

### 2.5. Data Collection Process

Data were independently extracted from each included report by two authors (L.-M.P. and A.-A.P.). In cases where discrepancies arose between their extractions, a third author (A.C.) was consulted to resolve the differences and reach consensus. No automation tools were used in the data collection process.

### 2.6. Outcomes Sought

The primary outcomes of interest were odds ratios (ORs) or hazard ratios (HRs) for each gene or SNP associated with acute or late skin side effects resulting from radiotoxicity. For each included study, all reported results compatible with this outcome domain were sought, including those from different analyses and time points. No restrictions were applied regarding the specific measure, timing, or type of analysis if the outcome was relevant to radiogenetic associations with skin toxicity.

### 2.7. Selected Articles

A total of 11 articles were ultimately included in the analysis. Of these, 6 studies reported data exclusively related to acute radiotherapy skin side effects, focusing on immediate reactions such as skin erythema, dry desquamation, moist desquamation, and acute radiodermatitis occurring within weeks or months following radiation therapy. Three studies were dedicated solely to late radiotherapy skin side effects, which typically manifest months or years after treatment and include fibrosis, telangiectasia, and other chronic radiation-induced complications. The remaining two studies provided data on both acute and late radiotherapy skin side effects, thus encompassing a broader spectrum of radiation-induced toxicities. 

Table 1 and Table 2 present the detailed characteristics of these studies, which are structured to provide comprehensive information on each study.

It is important to note that Table 1 and Table 2 do not duplicate the two studies conducted by Harkeran K. Jandu et al., 2023 [12] and Adam J. Webb et al., 2022 [16]. Instead, Table 1 presents the data related to acute radiotherapy skin side effects, while Table 2 includes information on late radiotherapy skin side effects. This separation ensures clarity and prevents redundancy when analyzing the findings.

Furthermore, additional details of the articles included in this review are presented in the Appendix A, structured as Table A1 and Table A2. These tables provide a more granular breakdown of the studies, categorized by their focus on acute or late skin radiotoxicity.

### 2.8. Excluded Studies and Reasons for Ineligibility

Thirteen studies were excluded during the process of assessing eligibility. The specific reasons for their exclusion are detailed in the Table A3.

### 2.9. Statistical Methods Applied

For statistical analysis, we used Jamovi, version 2.6.17.0 for MacBook, applying the random-effects model (effect sizes and standard errors) to account for heterogeneity between studies.

The random-effects model was applied separately to studies focusing on acute and late radiotherapy side effects. The results and their interpretation are presented in the following sections.

### 2.10. Bias Assessment

We assessed bias using Version 2 of the ROBINS-I tool from Cochrane Methods Bias, launched on 22 November 2024. Two authors, L.-M.P. and A.-A.P., independently evaluated the risk of bias for each included study, following the official ROBINS-I risk of bias tool guidelines. A third author, A.C., was consulted in cases where disagreements arose between the initial reviewers to enhance the accuracy and reliability of the bias assessment process.

The ROBINS-I tool was applied separately to studies focusing on acute and late radiotherapy side effects. The results and their interpretation are presented in the following sections.

## 3. Results

### 3.1. Results of the Assessment of Bias

We used the ROBINS-I risk of bias tool to assess the risk of bias for all articles included in this study. This tool enabled a systematic evaluation of potential biases across various domains, ensuring the reliability and validity of the findings. The summary of the ROBINS-I risk of bias assessment is presented in Section 3.1. At the same time, the detailed justifications for each domain of each article are provided in Appendix B, specifically in Table A4 and Table A5 for studies assessing acute and late radiotherapy-induced skin effects, respectively. In the following paragraphs, we will present the bias assessment results separately for the articles included in the analysis of genetic markers associated with acute radiotherapy-induced skin effects and the analysis of genetic markers associated with late radiotherapy-induced skin effects. This structured approach aims to provide a comprehensive understanding of the quality of the evidence about each category of radiotherapy-induced skin effects.

Each cell in the figure is color-coded based on the level of bias: green indicates a low risk of bias, yellow indicates a moderate risk of bias, and red indicates a serious risk of bias. Studies are listed in rows, while bias domains are represented by columns, culminating in an overall judgment in the final column.

**Figure 2:**
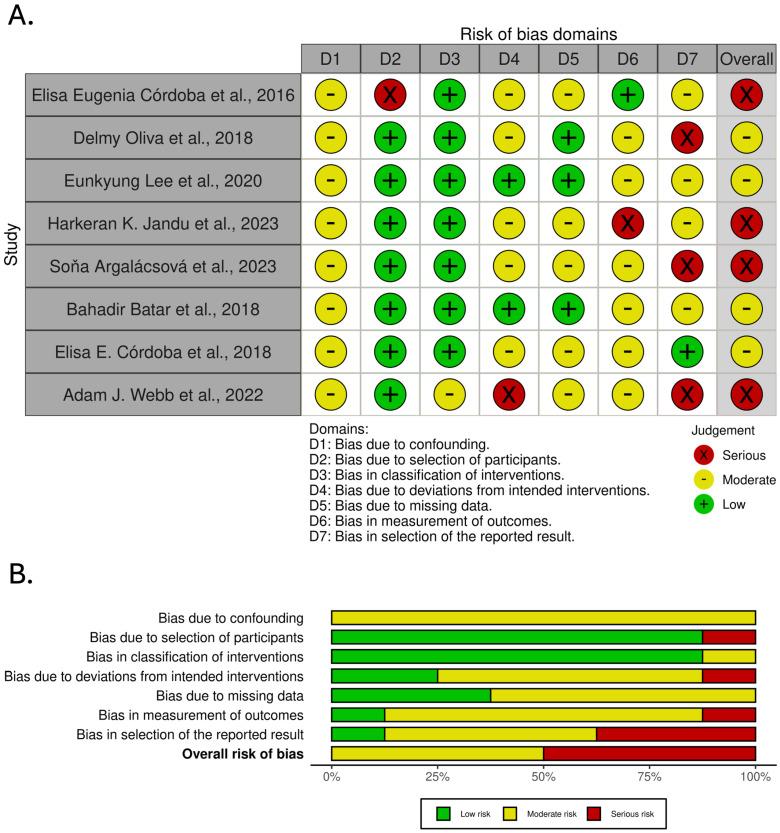
Risk of bias assessment across studies included in the analysis of genetic markers associated with acute radiotherapy-induced skin side effects. (**A**). traffic light plot. (**B**). summary plot [9,10,11,12,13,14,15,16].

Figure 2 presents the results of the risk of bias assessment performed using the ROBINS-I tool for studies included in the analysis of genetic markers associated with acute radiotherapy-induced skin side effects.

**Figure 3:**
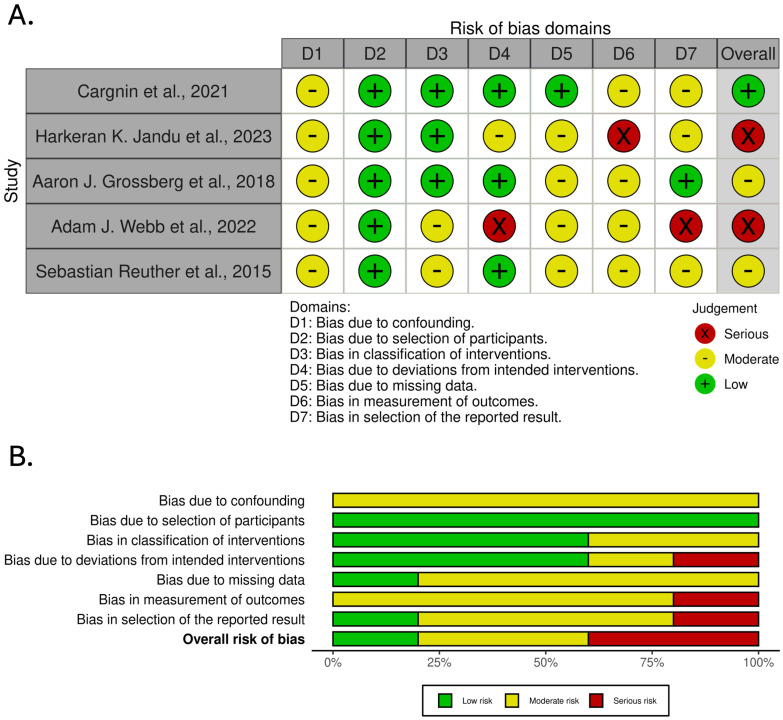
Risk of bias assessment across studies included in the analysis of genetic markers associated with late radiotherapy-induced skin side effects. (**A**). traffic light plot. (**B**). summary plot [12,16,17,18,19].

Figure 3 presents the results of the risk of bias assessment performed using the ROBINS-I tool for studies included in the analysis of genetic markers associated with late radiotherapy-induced skin side effects.

### 3.2. Impact of Excluding High-Risk Studies on Meta-Analysis Results

The majority of the articles included in this meta-analysis were found to have a moderate or high overall risk of bias based on the ROBINS-I risk of bias assessment. We performed sensitivity analyses by removing studies that were found to have a high risk of bias and comparing the outcomes with those obtained when all studies were included in order to assess the impact of study quality on our findings with and without these high-risk studies; thereby, pooled analyses were conducted for skin effects brought on by acute and late radiation therapy. To illustrate how effect sizes, heterogeneity, and statistical power may be impacted by the inclusion or exclusion of high-risk trials, we provide a succinct comparison of these two strategies in the paragraphs that follow.

When high-risk studies were excluded, the heterogeneity for late side effects decreased marginally, with an I^2^ of 92.75% as opposed to 99.45% when all studies were included. Despite this, the I^2^ still showed significant heterogeneity. Reduced robustness was also reflected in the fail-safe N, which dropped significantly from 30,445 (all trials) to 721 (high-risk studies excluded). After excluding high-risk studies, the pooled OR rose to 1.97 (95% CI: 1.181–2.756) from 1.44 (95% CI: 1.067–1.803) for all studies. This resulted in a broader CI and, thus, more uncertainty.

The tendency for acute side effects was comparable. After excluding high-risk studies, the I^2^ decreased from 99.3% (all studies) to 97.18%, which is still a decrease but still suggests extremely high heterogeneity. While the OR rose from 1.53 (95% CI: 1.107–1.945) to 1.83 (95% CI: 1.005–2.673), the fail-safe N decreased from 27,989 to 2150. This resulted again in a broader CI and, thus, more uncertainty.

We decided to present and discuss further the pooled meta-analysis containing all studies in the next sections due to the persistently high heterogeneity, the decreased statistical power, and the wider CIs seen when eliminating high-risk studies. Even when studies with a larger risk of bias are included, this method yields more consistent values and higher statistical power.

The following discussion section will explore the underlying factors contributing to the high heterogeneity observed in the meta-analysis results.

### 3.3. Pooled Analysis of Genetic Markers Associated with Radiotherapy-Induced Skin Acute Effects

This subsection presents the pooled analysis results assessing the association between genetic markers and skin acute effects of radiotherapy in breast cancer patients. The analysis was performed using a random-effects model to account for potential heterogeneity across studies. Table 1 above presents a short description of the included studies. For more characteristics of the included studies, please consult Table A1.

The pooled OR from the random-effects model is 1.53, with a 95% CI of 1.107 to 1.945 and a highly significant *p*-value of <0.001.

The heterogeneity statistics indicates substantial heterogeneity with an I^2^ of 99.3%, a Q-value of 180.714 (*p* < 0.001), a Tau value of 1.069, and a Tau^2^ value of 1.142.

**Figure 4:**
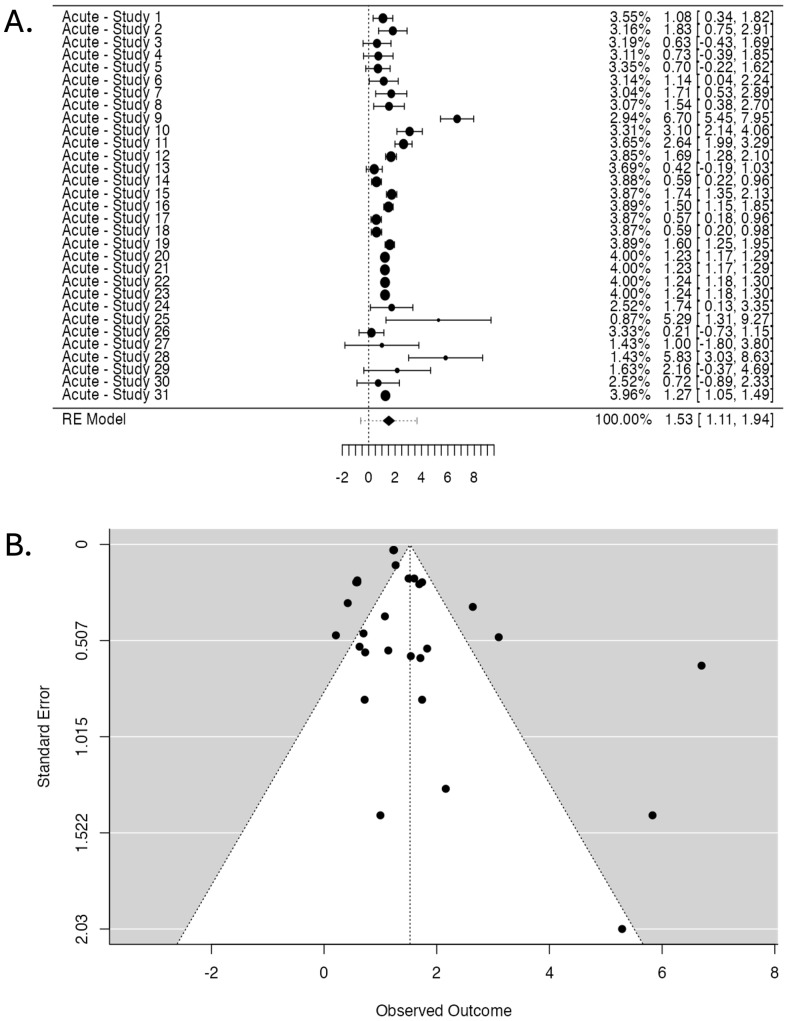
Studies investigating radiotherapy-induced skin acute effects. (**A**). Forest Plot displaying the pooled OR and 95% CI. The studies are listed on the left side, providing their labels (see Table 1 for labels), while the right side presents the corresponding weight (%), OR, and CIs for each study (the numbers in brackets). The larger points indicate a more significant influence on the overall pooled estimate due to larger sample sizes or higher precision. The central diamond at the bottom of the plot represents the pooled OR and 95% CI. (**B**). Funnel Plot assessing publication bias displays observed OR (X-axis) against standard error (Y-axis). Each point represents a study, with more extensive studies appearing at the top. The dotted lines form the expected distribution range under no publication bias.

The Forest Plot (Figure 4A) illustrates individual study ORs and 95% CIs. A central diamond represents the overall pooled estimate, which confirms the association’s significance. The Funnel Plot (Figure 4B) assesses publication bias. Studies are distributed asymmetrically, indicating a potential bias in the reported results.

The publication bias assessment shows a fail-safe N of 27,989 (*p* < 0.001), suggesting that many negative studies would be required to nullify the observed effect. However, Kendall’s Tau test did not reach statistical significance (0.199, *p* = 0.121). In contrast, Egger’s Regression Test demonstrated significant asymmetry (*p* = 0.012).

The 95% CI for the TOST procedure ranged from 1.174 to 1.877, while the corresponding Z-test 95% CI ranged from 1.107 to 1.945. The Z-values for the lower and upper bounds are 9.477 (*p* < 0.001) and 4.799 (*p* = 1.00), respectively, confirming a significant equivalence.

These findings provide an overview of the pooled analysis of studies investigating the association between genetic markers and radiotherapy-induced acute effects. The following sections will discuss the further interpretation and implications of these results.

### 3.4. Pooled Analysis of Genetic Markers Associated with Radiotherapy-Induced Skin Late Effects

This subsection presents the pooled analysis results assessing the association between genetic markers and the late skin effects of radiotherapy in breast cancer patients. The analysis was performed using a random-effects model to account for potential heterogeneity across studies. Table 2 above presents a short description of the included studies. For more characteristics of the included studies, please consult Table A2.

The pooled OR from the random-effects model is 1.44, with a 95% CI of 1.067 to 1.803 and a highly significant *p*-value of <0.001.

The heterogeneity analysis revealed substantial heterogeneity with an I^2^ of 99.45%, a Q-value of 170.026 (*p* < 0.001), a Tau value of 0.727, and a Tau^2^ value of 0.5282.

**Figure 5:**
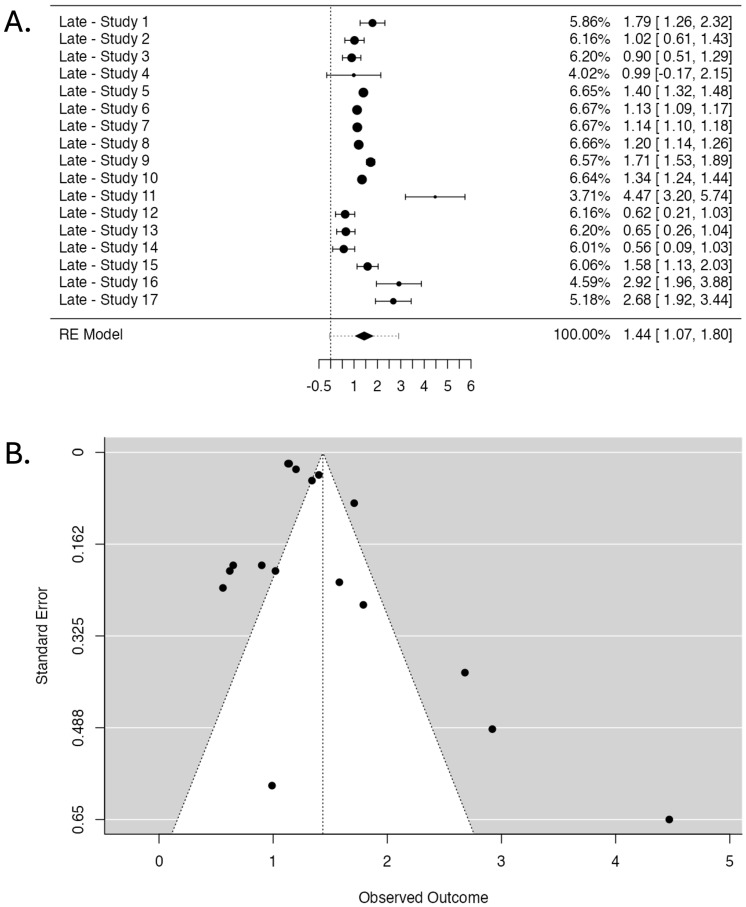
Studies investigating radiotherapy-induced late skin effects. (**A**). Forest Plot displaying the pooled OR and 95% CI. The studies are listed on the left side, providing their labels (see Table 2 for labels), while the right side presents the corresponding weight (%), OR, and CIs for each study (the numbers in brackets). The larger points indicate a more significant influence on the overall pooled estimate due to larger sample sizes or higher precision. The central diamond at the bottom of the plot represents the pooled OR and its 95% CI. (**B**). Funnel Plot assessing publication bias displays observed OR (X-axis) against standard error (Y-axis). Each point represents a study, with more extensive studies appearing at the top. The dotted lines form the expected distribution range under no publication bias.

The Forest Plot (Figure 5A) illustrates individual study ORs and 95% CIs. A central diamond represents the overall pooled estimate, which confirms the association’s significance. The Funnel Plot (Figure 5B) assesses publication bias. Studies are distributed asymmetrically, indicating a potential bias in the reported results.

The fail-safe N test yielded a high value of 30,445 (*p* < 0.001). However, Kendall’s Tau test did not reach statistical significance (*p* = 0.107). In contrast, Egger’s Regression Test demonstrated significant asymmetry (*p* = 0.004).

The results of the Two One-Sided Tests (TOST) procedure show statistically significant lower bound equivalence (Z-value = 10.331, *p* < 0.001) and non-significant upper bound equivalence (Z-value = 4.983, *p* = 1.00). The 95% CI for the TOST procedure ranges from 1.127 to 1.744, while the corresponding Z-test 95% CI ranges from 1.067 to 1.803.

These findings provide an overview of the pooled analysis of studies investigating the association between genetic markers and radiotherapy-induced late effects. The following sections will discuss the further interpretation and implications of these results.

### 3.5. Statistically Significant Genetic Markers Identified

This meta-analysis consolidates current evidence linking genetic variants with radiotherapy-induced skin toxicities in breast cancer patients, highlighting a multifaceted genetic landscape. Their relevance and future importance will be discussed further in the Discussions section.

As summarized in Table 3 and Table 4, this meta-analysis identified several genes (SNPs) that showed statistically significant associations with acute or late skin toxicities induced by radiotherapy in breast cancer patients. These findings are significant as they highlight potential predictive biomarkers that could guide future personalization of radiotherapy protocols.

A complicated and multivariate genetic landscape is revealed by this meta-analysis, which summarizes the most recent data on the relationship between genetic variations and skin toxicities caused by radiation therapy in patients with breast cancer. Polymorphisms in DNA repair genes (e.g., XRCC2, ERCC2, and ERCC1) and inflammatory mediators like IFNG were most linked to acute adverse effects, including erythema, desquamation, and oedema [10,11,14].

Furthermore, among other SNPs, the presence of XRCC2 (rs2040639), ATM (rs61915066), TGFB1 (C−509T), PER3 (rs2087947), and PAX7 (rs643644) was significantly linked to an increased risk of developing side effects, such as fibrosis, oedema, fibrosis, telangiectasia, lymphedema, erythema, and desquamation [10,11,12,16,18].

Significantly, some SNPs, such as ATM (rs61915066) and PER3 (rs2087947), were linked to both acute and late deleterious effects, suggesting a perhaps more extensive function in regulating individual radiosensitivity [16].

The variety of biological pathways that have been implicated—such as those that control immunological responses, DNA repair, and chronobiology—highlights the intricacy of host–radiation interactions and emphasizes the necessity of integrative, customized predictive models. The development of genetically guided radiation procedures that maximize therapeutic efficacy while reducing long-term damage is made possible by these discoveries.

## 4. Discussion

This section provides a full overview of our meta-analysis’ important findings and implications. We begin by looking at the link between genetic markers and acute and late radiotherapy-induced cutaneous toxicities in breast cancer patients. This is followed by an investigation into the clinical significance and future possibilities of the identified genetic variations. We then discuss the importance of genomic variables in defining individual radiosensitivity, as well as the potential for constructing predictive models to guide individualized radiotherapy. Finally, we explore how methodological discrepancies between the included studies affect the feasibility, validity, and generalizability of our meta-analysis findings in real-world clinical settings.

### 4.1. Discussion of Radiotherapy-Induced Skin Acute Effects and Associated Genetic Markers

The current study aimed to assess the association between genetic markers and radiotherapy-induced skin acute effects in breast cancer patients through a pooled analysis using a random-effects model. The findings demonstrated a pooled OR of 1.53 with a 95% CI of 1.107 to 1.945 and a highly significant *p*-value of <0.001. This suggests a 53% increased risk of developing acute radiotherapy-induced skin effects associated with the presence of specific genetic markers. The significance of this association, supported by a Z-value of 7.14, suggests that the results are robust under the statistical model applied.

However, the heterogeneity statistics indicate substantial variability among the included studies, with an I^2^ value of 99.3% and a Q statistic of 180.714 (*p* < 0.001). This high degree of heterogeneity suggests that the differences observed across studies are likely not due to random chance but instead arise from genuine variations in study characteristics, methodologies, and quality. Such significant heterogeneity implies that caution must be exercised when interpreting the pooled OR, as it may not represent a consistent effect across all studies.

The ROBINS-I risk of bias assessment (Figure 2) further contextualizes these findings by providing insight into the methodological quality of the studies included in the meta-analysis. The assessment revealed a predominant presence of moderate-to-serious risks of bias, particularly in domains related to D1, D5, and D7. The presence of a serious risk of bias in some studies, particularly related to confounding and deviations from intended interventions (D4), likely contributes to the high heterogeneity detected by the random-effects model.

Comparing the findings of the ROBINS-I tool to the heterogeneity statistics, it becomes apparent that the studies rated as having serious risk of bias in key domains likely contribute disproportionately to the overall heterogeneity. For example, studies with a serious risk of bias in D1 (Confounding) may yield higher OR estimates due to inadequate adjustment for potential confounding factors, such as age, BMI, or radiotherapy dose variations. Similarly, studies with a serious risk of bias in D5 (missing data) may have compromised statistical power or biased effect estimates, further exacerbating heterogeneity.

Furthermore, the Forest Plot (Figure 4A) shows considerable variability in the effect sizes reported by individual studies, which correlates well with the ROBINS-I tool’s findings of diverse risk of bias across studies. Studies with higher methodological rigor (i.e., those rated with low or moderate risk of bias) tend to have effect estimates clustered more closely around the pooled OR, while studies with a higher risk of bias display wider CIs and more extreme ORs. This observation underscores the importance of considering study quality when interpreting pooled estimates from meta-analyses.

The publication bias assessment further complicates the interpretation of the results. While the fail-safe N of 27,989 (*p* < 0.001) indicates that a substantial number of null studies would be needed to negate the observed effect, the Egger’s Regression Test (*p* = 0.012) suggests the presence of publication bias. This is corroborated by the Funnel Plot (Figure 4B), which shows asymmetry, indicating that smaller studies with null or negative results may be underreported or omitted. Such bias can lead to overestimating the pooled effect size, especially when combined with the influence of studies with a high risk of bias identified through the ROBINS-I tool.

It is also notable that studies with a serious risk of bias in domains such as D6 (measurement of outcomes) and D7 (selection of the reported result) may introduce systematic errors that directly affect the observed associations. Poor outcome measurement or selective reporting can artificially inflate the effect sizes, contributing to both heterogeneity and publication bias. The ROBINS-I tool effectively highlights these issues, providing a framework for understanding how study quality may influence the pooled estimate.

The Two One-Sided Tests (TOST) for equivalence testing further validate the robustness of the findings to some extent. While the lower bound Z-value (9.477, *p* < 0.001) confirms a statistically significant association, the upper bound Z-value (4.799, *p* = 1.00) suggests limited precision in the higher end of the effect estimate. This is consistent with the high heterogeneity observed and may also reflect the influence of studies with a higher risk of bias.

The ROBINS-I tool’s findings, particularly those indicating a serious risk of bias in D1, D4, D5, D6, and D7, are highly relevant to interpreting the meta-analysis results. The high heterogeneity detected by the random-effects model is partly driven by studies with an elevated risk of bias, as identified through the ROBINS-I assessment. Furthermore, as suggested by Egger’s Regression Test and the Funnel Plot, the publication bias reinforces the need for a cautious interpretation of the pooled OR.

Future research should prioritize including high-quality studies with robust methodologies to minimize bias in confounding, missing data, and outcome measurement. Additionally, efforts should be made to ensure that all relevant studies, including those with negative findings, are published to mitigate publication bias. Adopting a more stringent approach to the risk of bias assessment and ensuring greater consistency in study methodologies will be essential to accurately determine the relationship between genetic markers and radiotherapy-induced acute effects.

### 4.2. Discussion of Radiotherapy-Induced Skin Late Effects and Associated Genetic Markers

The present study aimed to evaluate the association between genetic markers and radiotherapy-induced skin late effects in breast cancer patients through a pooled analysis using a random-effects model. The results indicated a pooled OR of 1.44 with a 95% CI of 1.067 to 1.803 and a statistically significant *p*-value of <0.001. This finding suggests a 44% higher risk of experiencing late radiotherapy-induced skin effects among individuals with specific genetic markers than their counterparts. The robustness of this association is further supported by a high Z-value of 7.65.

Despite the statistical significance, the heterogeneity statistics reveal high variability across the included studies, with an I^2^ value of 99.45% and a Q statistic of 170.026 (*p* < 0.001). This level of heterogeneity implies that the observed differences are not solely due to random variation but are likely influenced by genuine discrepancies in study designs, genetic marker assessment methodologies, and patient populations. Such a high heterogeneity indicates that the pooled estimate may not uniformly apply to all studies included in the analysis.

The ROBINS-I risk of bias assessment (Figure 3) offers critical insights into the quality of the studies contributing to the meta-analysis. Notably, many studies exhibited moderate-to-serious risks of bias, particularly in areas such as D1 (confounding) and D5 (missing data). The high risk of bias in these domains is especially concerning, as inadequate control for confounding variables or incomplete data could substantially skew the observed associations.

A serious risk of bias in several studies likely contributes to the substantial heterogeneity detected by the random-effects model. Specifically, studies with a serious risk of bias in D1 (confounding) may exhibit exaggerated effect estimates if relevant confounders, such as age, genetic background, radiation dose, or therapeutic interventions, are not adequately controlled. Furthermore, D5 (missing data) presents another critical source of bias, as studies with substantial missing data may yield misleading results due to reduced statistical power or biased effect estimates.

The Forest Plot (Figure 5A) illustrates the considerable variability in effect sizes reported by individual studies. The plot demonstrates a wide range of ORs, which correlates well with the findings from the ROBINS-I tool, where studies with higher risks of bias tend to have wider CIs and more extreme effect sizes. The central diamond representing the pooled OR indicates a statistically significant association. However, the width of the CI reflects the high heterogeneity and the influence of methodological inconsistencies across studies.

The publication bias assessment presents additional challenges to the interpretation of these findings. The fail-safe N test, yielding a value of 30,445 (*p* < 0.001), suggests that a substantial number of null studies would negate the observed effect. However, Egger’s Regression Test (*p* = 0.004) indicates the possibility of publication bias. The Funnel Plot (Figure 5B) displays a noticeable asymmetry, particularly with studies clustering more densely on one side, suggesting that smaller studies with null or negative findings may be underreported. This could artificially inflate the pooled estimate, especially when combined with the influence of studies identified as having serious bias by the ROBINS-I assessment.

The Two One-Sided Tests (TOST) for equivalence testing reveal further complexities in interpreting the pooled effect. While the lower bound Z-value (10.331, *p* < 0.001) confirms a statistically significant association, the upper bound Z-value (4.983, *p* = 1.00) indicates limited precision in estimating the upper range of the effect size. The CIs for the TOST procedure (1.127 to 1.744) and the Z-test (1.067 to 1.803) further emphasize this lack of precision.

Comparing the ROBINS-I findings to the statistical analysis underscores several important considerations. Studies identified as having a serious risk of bias in D1, D5, and D6 (measurement of outcomes) likely contribute disproportionately to the high heterogeneity observed. Additionally, poor outcome measurement or selective reporting (D6 and D7) could influence the robustness of the pooled estimate by introducing systematic errors. Studies with a higher risk of bias often report more extreme ORs, likely due to inadequate confounder control or missing data, which may inflate the overall pooled effect size.

Moreover, the apparent asymmetry observed in the Funnel Plot (Figure 5B) could be attributed to publication bias and underlying heterogeneity driven by differences in methodological quality across studies. Studies rated with a low or moderate risk of bias generally show more consistent effect estimates, while those with serious bias tend to deviate substantially from the pooled estimate.

Taken together, the high degree of heterogeneity, potential publication bias, and presence of methodological flaws identified by the ROBINS-I tool suggest that the observed association between genetic markers and radiotherapy-induced late effects should be interpreted with caution. While the findings are statistically significant, the robustness of these results is undermined by inconsistencies in study quality and potential bias.

Future research should focus on improving study design consistency, minimizing bias through rigorous methodologies, and ensuring the inclusion of all relevant studies to address potential publication bias. Efforts to standardize genetic marker assessment protocols and improve data completeness will be crucial in enhancing the reliability of future meta-analyses investigating genetic predictors of radiotherapy-induced late effects.

### 4.3. Pathway-Based Analysis of Radiogenomic Markers in Skin Toxicity

Radiogenomic research has considerably deepened our understanding of individual variability in radiation-induced toxicity. While classical radiobiological models have primarily focused on dosimetry and tumor radiosensitivity, there is now compelling evidence that hosts genetic architecture plays a pivotal role in modulating tissue response to ionizing radiation. The intricate interactions between DNA damage response (DDR), circadian regulation of cellular physiology, oxidative stress, and detoxification systems and immune–inflammatory signaling cascades result in radiation-induced toxicity, a complex, multifactorial pathological process [1,2,20,21,22].

Our findings corroborate a growing body of literature identifying SNPs and broader polygenic profiles as potent modifiers of acute and late radiation toxicity phenotypes. In the following subsections, we discuss the molecular pathways involving the SNPs identified through our systematic investigation in this meta-analysis.

#### 4.3.1. DNA Repair Genes and Radiotherapy Toxicity

Radiotherapy-induced skin toxicity—both acute and late—results from a complex interplay between genetic variants in DNA damage response (DDR) genes and the cellular mechanisms governing DNA repair, cell cycle control, and apoptosis. Ionizing radiation (IR), a potent genotoxic agent, causes various DNA lesions, among which double-strand breaks (DSBs) are the most lethal. The efficient repair of these lesions is essential for maintaining genomic stability and mitigating radiation toxicity [23].

Our meta-analysis identified statistically significant and non-significant associations between DDR gene polymorphisms and acute skin reactions following radiotherapy. Delmy Oliva et al. (2018) [10] found that XRCC2 (rs2040639), a critical component of the homologous recombination (HR) pathway, increases the risk of acute skin reactions such as itching and burning. This aligns with mechanistic insights showing that XRCC2 interacts with RAD51B and RAD51D to stabilize RAD51 nucleofilaments during HR, essential for high-fidelity repair during the S and G2 phases [24]. Polymorphisms in XRCC2 can impair filament stabilization, reducing repair efficacy and enhancing radiosensitivity [25].

Similarly, Eunkyung Lee et al. (2020) [11] reported associations between increased acute toxicity and polymorphisms in ATM (rs61915066), CHEK1 (rs11220184), ERCC2 (rs1799786), and RAD51C (rs405684). ATM is a master regulator of DDR that detects DSBs and activates downstream effectors like p53, CHK2, and BRCA1, enforcing checkpoint control and determining DNA repair pathway choice [26]. Impairments in ATM signaling due to SNPs may weaken these critical protective responses, predisposing tissues to radiation damage. Similarly, RAD51C, like XRCC2, is vital for HR, and polymorphisms that diminish its activity can reduce repair fidelity and heighten acute skin toxicity [27].

Interestingly, the same study also identified protective roles for ERCC2 (rs60152947 and rs10404465) and RAD51C (rs302877) [11], consistent with findings that certain SNPs may enhance repair efficiency or modulate transcriptional responses favorably. ERCC2 (also known as XPD) participates in nucleotide excision repair (NER), and some polymorphisms may confer more efficient lesion recognition or excision [28].

Contrasting results were seen in Soňa Argalácsová et al. (2023) [13], who reported no statistically significant associations between BRCA1, BRCA2 (OR = 1.74, *p* = 0.50), or a group of DDR genes including CHEK2, ATM, PALB2, RAD51C, RAD51D, BARD1, TP53, and FANCM (OR = 5.29, *p* = 0.41) and acute dermatitis or lymphedema. However, given BRCA1 and BRCA2’s central roles in HR through interactions with PALB2 and RAD51 [29,30], these findings may reflect the complexity of compensatory pathways and environmental influences.

Further supporting a protective genetic background, Bahadir Batar et al. (2018) [14] identified ERCC1 (rs3212986) as a significant protective factor (OR = 0.21, *p* < 0.001) against acute skin reactions. ERCC1 is involved in both NER and interstrand crosslink repair; variants that enhance its function may improve the repair of bulky DNA adducts induced by IR [31]. On the other hand, Elisa E. Córdoba et al. (2018) [15] did not find a statistically significant association between ATM (rs1801516) and severe radiodermatitis (OR = 0.72, *p* = 0.68), emphasizing that not all polymorphisms in key genes translate into clinically measurable effects.

Our article also explored associations between genetic variants and late skin toxicity. Cargnin et al. (2021) [17] found that TP53 (rs1042522) was a risk factor for subcutaneous fibrosis and/or telangiectasia (OR = 1.79, *p* = 0.028). This SNP results in an Arg-to-Pro substitution at codon 72, which may alter p53’s apoptotic potential. Given p53’s centrality in controlling cell cycle arrest and apoptosis following IR-induced damage [32], impaired function may promote fibrotic remodeling due to insufficient clearance of damaged cells.

However, the same study found no significant association for ERCC2 (rs1052555 and rs13181) or LIG1 (rs7246696) [17]. ERCC2’s role in NER may be less crucial during the late fibrotic phase, while LIG1, involved in Okazaki fragment joining during replication, may not be rate-limiting for late tissue remodeling.

Sebastian Reuther et al. (2015) [19] observed that a pooled group of DNA repair genes was significantly associated with increased risk of subcutaneous fibrosis (OR = 2.92, *p* = 0.023), reinforcing the multifactorial nature of late toxicity and the combined effect of multiple low-penetrance variants. Late side effects often reflect chronic inflammation, vascular damage, and fibrogenesis, processes intimately linked with persistent DNA damage and inadequate repair, particularly by NHEJ and HR [33,34].

Altogether, these findings underscore the importance of genetic variants in DDR genes—particularly those involved in homologous recombination (XRCC2 and RAD51C), checkpoint control (ATM, CHEK1, and TP53), and excision repair pathways (ERCC1 and ERCC2)—in modulating individual susceptibility to both acute and late radiation-induced skin toxicity. These polymorphisms likely influence the efficiency, accuracy, and coordination of DNA repair processes following IR, ultimately affecting the balance between tissue regeneration and pathological remodeling. Understanding these associations paves the way for personalized radiotherapy approaches, incorporating genetic risk profiling to optimize therapeutic outcomes and minimize adverse effects.

#### 4.3.2. Circadian Rhythm and Radiotherapy Toxicity

Our meta-analysis identified several statistically significant associations between circadian rhythm-related genes and radiotherapy-induced skin toxicities. Regarding acute side effects, Adam J. Webb et al. (2022) [16] found that the PER3 (rs2087947) variant is associated with an increased risk for acute erythema (OR = 1.27, *p* = 0.02). In terms of late skin side effects, the same study [16] reported that CLOCK (rs1801260) (OR = 0.62, *p* < 0.01), PER3 (rs2087947) (OR = 0.65, *p* = 0.04), and RASD1 (rs11545787) (OR = 0.56, *p* = 0.02) are protective factors for breast atrophy two years post-radiotherapy.

These findings align with emerging molecular insights into the role of circadian rhythms in modulating DNA repair, cell cycle regulation, and response to ionizing radiation. Circadian rhythms are regulated by transcription–translation feedback loops involving core clock genes such as CLOCK, BMAL1, PER1, PER2, and CRY, which control the rhythmic expression of a wide array of target genes, including those involved in the DDR [35].

PER1 has been shown to enhance DNA double-strand break (DSB) repair by physically interacting with ATM and CHK2, promoting phosphorylation cascades that activate p53-dependent cell cycle arrest and apoptosis after radiation exposure. Overexpression of PER1 sensitizes cells to radiation, while loss of PER2 delays CHK2 activation and compromises repair, potentially leading to increased genomic instability or radiation resistance. These mechanisms support the finding that the PER3 variant may exacerbate acute erythema, as reduced or altered PER function could diminish effective damage signaling and apoptotic clearance of irradiated cells [36].

Similarly, the CLOCK-BMAL1 complex orchestrates circadian control of the G1/S and G2/M cell cycle checkpoints, partly through regulation of WEE1 and HIF-1α expression. Loss of BMAL1 has been shown to impair p53 activation following radiation and weaken checkpoint integrity, particularly in keratinocytes, where it leads to a build-up of UVB-induced DNA lesions [37]. Given CLOCK’s role in DDR and oxidative stress responses, the protective association of the CLOCK (rs1801260) polymorphism with late tissue damage [16] might reflect enhanced transcriptional regulation of DNA repair and hypoxia-related genes, limiting chronic radiation-induced fibrosis or atrophy.

RASD1, another gene implicated in our review [16], encodes a Ras-related signaling protein that integrates signals from the circadian system and cellular stress pathways. RASD1 is rhythmically regulated and plays a role in modulating glucocorticoid receptor activity, which intersects with radiation responses and inflammatory signaling. Its polymorphism (rs11545787) being protective against late atrophy supports the hypothesis that intact circadian regulation mitigates prolonged tissue damage and enhances post-radiotherapy recovery [36,37].

Furthermore, chronoradiotherapy—delivering treatment at biologically optimized times—emerges as a promising strategy to improve therapeutic outcomes. Preclinical evidence shows that radiation sensitivity fluctuates over a 24-h cycle, with skin DNA repair activity lowest in the early morning and peak radiosensitivity occurring at specific circadian phases [36,38]. The CLOCK-BMAL1 complex and its downstream targets (e.g., PERs, CRYs, TIMELESS, and WEE1) modulate the cell cycle and DDR checkpoints, creating windows of heightened or reduced susceptibility to radiation damage [38].

Importantly, the study by Webb et al. [16] involving the REQUITE cohort provided direct evidence of how circadian timing interacts with genetic background to influence radiotherapy outcomes. While time of day of treatment did not significantly affect acute erythema risk, it had a strong interaction with genetic variants for late tissue toxicity. For instance, patients with certain CLOCK, PER3, or RASD1 risk alleles could reduce their likelihood of developing breast atrophy from 70% to 33% simply by receiving radiotherapy in the morning rather than the afternoon [35].

This supports a tailored approach to radiotherapy scheduling that considers not only the molecular clock but also patient-specific genetic profiles. Circadian disruption—due to shift work, irregular feeding, or metabolic disorders—has been linked to reduced treatment efficacy and increased toxicity, underscoring the need to preserve circadian homeostasis during cancer care [16]. Strategies such as time-restricted feeding and metabolic interventions could further align the DDR and cellular metabolism with optimal treatment windows.

The integration of circadian biology and genetic screening into radiotherapy protocols holds promise for reducing adverse effects and enhancing therapeutic efficacy. The PER3, CLOCK, and RASD1 polymorphisms identified in our systematic review [16], in conjunction with mechanistic studies of circadian regulation of DDR [36,38], highlight the translational potential of personalized chronoradiotherapy.

#### 4.3.3. Oxidative Stress Pathway in Radiation Skin Toxicity

Ionizing radiation (IR), a cornerstone of cancer therapy, exerts its biological effects not only through direct DNA damage but also through the generation of reactive oxygen species (ROS) via water radiolysis. These ROS—superoxide anions, hydrogen peroxide, and hydroxyl radicals—can induce oxidative modifications in nucleic acids, proteins, and lipids, leading to cellular injury, mitochondrial dysfunction, and, ultimately, tissue toxicity [39,40]. The antioxidant defense system, particularly enzymes like glutathione S-transferases (GSTs), superoxide dismutases (SODs), and nitric oxide synthases (NOS), plays a vital role in neutralizing ROS and mitigating radiation-induced damage.

In the context of acute skin side effects, our meta-analysis did not identify statistically significant associations between polymorphisms in several ROS pathway genes and increased or decreased risk of radiation-induced skin toxicity. Specifically, Elisa Eugenia Córdoba et al. (2016) [9] found no significant associations for GSTP1 (rs1695) (OR = 1.08, *p* = 0.88, and OR = 1.83, *p* = 0.27), NOS3 (rs1799983) (OR = 1.14, *p* = 0.82, and OR = 0.70, *p* = 0.45), GSTA1 (rs3957356) (OR = 1.71, *p* = 0.36, and OR = 1.54, *p* = 0.46), and SOD2 (rs4880) (OR = 0.63, *p* = 0.39, and OR = 0.73, *p* = 0.57), failing to establish either risk or protective roles in acute toxicity outcomes.

The absence of significant findings may be due to the multifactorial and redundant nature of the antioxidant system. For instance, GSTP1, beyond its role in detoxifying electrophilic compounds via glutathione conjugation, also modulates stress kinase pathways like JNK and TRAF2, influencing apoptosis and cellular proliferation [39,41]. A well-known polymorphism, GSTP1 Ile105Val, is associated with reduced enzymatic efficiency, which may explain susceptibility to radiation pneumonitis and possibly skin toxicity in other settings. SOD2, a key mitochondrial enzyme, converts superoxide radicals into hydrogen peroxide and influences mitochondrial function and apoptosis through cytochrome c release [42].

In contrast, regarding late skin side effects, our analysis did reveal statistically significant associations. Sebastian Reuther et al. (2015) [19] reported that a gene group from the ROS pathway—comprising GSTP1, SOD2, NQO1, NOS3, and XDH—was significantly associated with increased risk of subcutaneous fibrosis (OR = 1.58, *p* = 0.045). This finding underscores the role of persistent oxidative stress and inadequate antioxidant defense in the development of late tissue fibrosis.

Prolonged ROS exposure post-radiation can lead to endoplasmic reticulum (ER) stress, activation of the unfolded protein response (UPR), and ferroptosis, a regulated cell death pathway driven by lipid peroxidation [43]. GSTP1 has also been implicated in modulating ferroptosis through glutathione (GSH) metabolism, while IR-induced inhibition of GPX4, a key enzyme preventing lipid peroxidation, promotes ferroptotic cell death [44]. This interplay among oxidative stress, ferroptosis, and immune–inflammatory pathways may explain the progression from acute injury to chronic fibrosis observed in some patients [41].

In summary, while individual polymorphisms in ROS pathway genes may not always yield statistically significant results in the context of acute toxicity, the cumulative effect of multiple gene variants likely contributes to long-term skin complications after radiotherapy. Future research should consider gene–gene and gene–environment interactions, as well as mechanistic studies on ferroptosis and ER stress responses, to better understand and mitigate radiation-induced late skin toxicities.

#### 4.3.4. Inflammatory Gene Polymorphisms in Radiation Skin Toxicity

Radiation-induced tissue damage triggers a complex cascade of inflammatory and immune responses that are integral to the development of both acute and chronic toxicities. This process begins with the release of damage-associated molecular patterns (DAMPs), which engage pattern recognition receptors and initiate sterile inflammation. Key pro-inflammatory cytokines such as tumor necrosis factor-alpha (TNF-α) and interleukin-6 (IL-6) play central roles in this response, coordinating the recruitment and activation of immune effector cells and shaping the extent of tissue injury. Elevated expression levels of TNF-α and IL-6 have been associated with increased severity in radiation-induced toxicities such as pneumonitis and fibrosis. Genetic polymorphisms in the promoter regions of these cytokine genes are known to modulate their expression, potentially altering the intensity of inflammatory responses and the risk of adverse effects [45,46].

However, our meta-analysis did not identify statistically significant associations between polymorphisms in these genes and acute skin toxicity outcomes. Specifically, Elisa E. Córdoba et al. (2018) [15] reported that TNF-α (rs1800629) (OR = 5.83, *p* = 0.22) and IL-6 (rs1800795) (OR = 2.16, *p* = 0.54) were not significantly associated with an increased risk of severe radiodermatitis (grades 3 and 4). These findings suggest that while these cytokines are biologically relevant to radiation-induced inflammation, the investigated genetic variants may not be strong or consistent predictors of acute skin toxicity, at least within the populations studied.

Further mechanistic insights underscore the broader immunological context of radiation injury. Radiation also leads to cytosolic accumulation of DNA fragments, activating the cyclic GMP-AMP synthase (cGAS)–stimulator of interferon genes (STING) pathway. This pathway promotes type I interferon responses and enhances innate immune activation [47,48]. Additionally, interferon-gamma (IFN-γ), produced by activated T cells and natural killer cells, can synergize with radiation to upregulate pro-apoptotic genes, potentially increasing collateral tissue damage. Polymorphisms in IFNG may further influence the magnitude of these immune responses.

Persistent inflammation can evolve into chronic tissue remodeling, driving fibroblast activation, extracellular matrix deposition, and fibrosis, a hallmark of late radiation toxicity [49,50]. Although our findings did not establish a clear genetic link between TNF-α or IL-6 variants and acute skin toxicity, the overall role of immune signaling in radiation response remains well supported and warrants further investigation across diverse cohorts and genetic backgrounds.

### 4.4. Relevance and Future Potential of Genetic Markers Identified

This meta-analysis consolidates current evidence linking genetic variants with radiotherapy-induced skin toxicities in breast cancer patients, highlighting a multifaceted genetic landscape. Our meta-analysis showed that patients carrying these mutations exhibited an estimated 53% higher risk for acute skin toxicities and 44% higher risk for late effects, as demonstrated by the pooled ORs derived from our statistical analysis.

The statistically significative SNPs identified in the literature (presented in Table 3 and Table 4), although promising, must be interpreted with caution. The associations found in the included studies must be replicated and validated through independent, large-scale, and multicenter investigations to confirm their utility as reliable biomarkers. This is essential to strengthen the scientific consensus and reduce the risk of false positives or overestimations caused by publication bias or methodological heterogeneity.

Importantly, while SNPs that exhibited statistically significant associations may be seen as more important, it is essential not to overlook those variants that did not reach statistical significance (see Table 1 and Table 2). These may also hold crucial information. Their lack of significance in their original studies could be attributed to underpowered sample sizes, differences in population genetics, or methodological limitations. Further studies may confirm these SNPs as truly non-significant or, conversely, as false negatives that will prove relevant upon reanalysis with improved data.

Collectively, both significant and non-significant SNPs identified through the systematic search of the literature contribute to the growing body of evidence needed to characterize the genetic landscape of radiotoxicity fully. This comprehensive understanding is pivotal because, in the future, radiotherapy protocols could be tailored based on a patient’s genetic profile. Even if clinical implementation remains a long-term goal, these insights already offer value in shaping predictive tools for identifying high-risk patients and designing preventive strategies—including pharmacological interventions or dose modulation—to reduce or preempt side effects [51,52,53].

Beyond individual variants, emerging data supports a polygenic risk score (PRS) framework. The REQUITE project, a prospective multicenter cohort study encompassing over 4400 patients, exemplifies the translational shift toward multifactorial risk models. Notably, patients in the highest PRS quartile for early adverse skin reactions (EASRs) had an 8.64-fold increased risk compared to the lowest quartile (*p* < 0.0001), reinforcing the concept that small-effect-size alleles, when aggregated, can provide substantial predictive power. Key contributors to these PRS include TGFβ1, ERCC2, ATM, and CHEK1 variants, which are involved in TGFβ-mediated fibrotic signaling, cell cycle checkpoint control, and nucleotide excision repair—all pivotal to radiation response [11,54].

This underscores the idea that while individual SNPs often have small effect sizes, their aggregated impact can yield substantial predictive power. In our meta-analysis, we adopted a similarly inclusive approach by presenting all statistically significant and non-significant ORs reported across studies. Some SNPs had modest ORs but were still considered relevant risk or protective factors; others showed stronger associations, with ORs ranging from 2 to 6. This comprehensive inclusion allows for a broader understanding of the genetic landscape influencing radiotherapy-induced skin toxicity. Aligning with the REQUITE findings, our pooled analysis revealed that patients with certain genetic variants have a 53% greater risk of developing acute skin toxicities and a 44% greater risk of experiencing late skin toxicities. These results support the potential of combining multiple SNPs, regardless of individual effect size, to develop more accurate and clinically useful predictive models for personalized radiotherapy planning.

Ultimately, this meta-analysis supports the integration of genetic data into oncological practice, representing a foundational step toward personalized radiotherapy and improved patient outcomes. Collectively, these findings contribute to a paradigm shift in radiation oncology—from population-based standardization to molecularly guided personalization. The incorporation of PRS, germline testing, immune profiling, and machine learning analytics holds the potential to stratify patients not only by their risk of toxicity but also by their expected therapeutic benefit, enabling treatment de-escalation or intensification as needed. While the path from genetic association to clinical application demands careful validation, and challenges such as variant interpretation, data privacy, and equitable access remain, the translational pipeline for radiogenomics is now well underway.

### 4.5. Impact of Methodological Differences Among Included Studies

Certain publications had a high risk of bias, according to the risk of bias assessment for the papers that were part of this meta-analysis. In the paragraphs that follow, we examine how the overall heterogeneity found in our analysis might have been influenced by the methodological differences among the chosen studies.

Notably, there was considerable variation in radiotherapy protocols. Although the average radiation dose across studies was approximately 50 Gy [9,10,11,12,13,14,15,16,17,18,19]. Furthermore, the assessment methods for skin side effects varied widely. A range of grading systems and criteria were used, including the Radiation Therapy Oncology Group (RTOG) criteria [9,10,15], Visual Analog Scale (VAS) [10], Oncology Nursing Society (ONS) scale [11], Common Terminology Criteria for Adverse Events (CTCAE) [12,13,16], Common Toxicity Criteria (CTC) [14], and the LENT-SOMA scale [17,18,19]. In addition, the wayside effects were defined, graded, and named differed between studies, adding further inconsistency.

The statistical methods used for analysis also varied, which may have influenced the reported outcomes and their comparability. Demographic variability was another factor, with the mean age of study populations ranging from 48.7 to 60.8 years [9,10,11,12,13,14,15,16,17,18,19].

Regarding study design, most included articles were observational in nature [9,10,11,13,14,15,18,19], with only a small number being case-control studies [12,16,17]. From our perspective, the inclusion of case-control studies likely had minimal impact on heterogeneity, as they are a subtype of observational studies and share many methodological characteristics.

The fact that all the included studies were retrospective is also crucial because this could add bias because of things like the use of previously recorded data, possible discrepancies in documentation, and the inability to account for confounding variables at the time of data collection [9,10,11,12,13,14,15,16,17,18,19]. When evaluating the pooled data, these retrospective components should be considered since they might have further increased variability.

A major source of heterogeneity in this meta-analysis is the wide range of methodological differences among the included studies. All the included studies are retrospective, which increases the possibility of bias brought on by flaws like unmeasured confounding and inadequate reporting. The choice to present the pooled analysis encompassing all studies was supported by the persistently high heterogeneity, decreased statistical power, and larger CIs that resulted from the exclusion of high-risk studies, as was previously mentioned in Section 3.2. Stronger statistical power and more consistent effect estimates were obtained with this method, even if higher-risk studies were included. The results of this meta-analysis reflect the well-known conclusion that methodological design differences among studies are frequently a more significant source of heterogeneity than the inclusion of high-risk studies alone.

### 4.6. Feasibility, Validity, and Generalizability of the Meta-Analysis Findings

The adequate number of relevant studies that examined the relationship between genetic variations and skin toxicities brought on by radiation therapy in patients with breast cancer made this meta-analysis possible. Enough similar data could be retrieved from many studies to allow for pooled analysis, even in the face of variations in methodology and outcome measurements.

We conducted sensitivity analyses by eliminating papers that the ROBINS-I tool determined to have a high overall risk of bias to evaluate the internal validity and robustness of our findings. The impact of study quality on effect sizes, heterogeneity (I^2^), and statistical power was revealed by comparing pooled outcomes with and without high-risk trials. Excluding high-risk studies resulted in wider CIs and much lower fail-safe N values, indicating fewer stable estimates and less statistical power, even if it also somewhat decreased heterogeneity. These trends held true for both immediate and delayed adverse effects. Even after removing high-risk studies, the heterogeneity persisted, which lends credence to the idea that methodological variation, not bias, is a major contributor to variance. Recognizing that all results must be evaluated considering underlying methodological limitations, the inclusion of all studies in the primary analysis was justified to maximize statistical power and produce more consistent estimates.

The included studies were conducted in a variety of countries, including Argentina, Sweden, the United States, France, Italy, Belgium, Spain, the United Kingdom, the Czech Republic, Turkey, and Germany, and represented a wide range of clinical settings, healthcare systems, and radiotherapy practices. This worldwide representation strengthens the external validity of our findings and matches them with real-world variations in breast cancer treatment. However, despite their geographical breadth, several studies did not provide thorough information on the ethnic composition of their participants, limiting our ability to assess the results’ relevance to ethnically varied patient groups. Additionally, because all investigations were retrospective, generalization to prospective or controlled clinical settings is limited. Differences in outcome definitions, toxicity grading scales, and data collection procedures complicate direct cross-study comparisons.

Nonetheless, the inclusion of real-world data from a variety of healthcare situations strengthens the therapeutic significance of our findings and highlights the potential of genetic markers in predicting radiotherapy-induced skin toxicities.

In summary, this meta-analysis was feasible due to the availability of sufficient comparable data across diverse studies. Sensitivity analyses validated the robustness of the findings, showing that methodological variability was a greater source of heterogeneity than bias alone. The inclusion of studies from multiple countries supports generalizability to real-world clinical settings, though limitations in demographic detail and retrospective designs suggest cautious interpretation.

## 5. Limitations

Although this meta-analysis provides valuable insights into the genetic factors influencing radiotherapy-induced skin toxicity in breast cancer patients, several limitations must be acknowledged. These limitations relate to the included studies’ methodological heterogeneity and the broader challenges inherent in radiogenomic research. Addressing these issues in future work will be essential for improving the reliability and clinical translation of genetic predictors.

1. High inter-study heterogeneity: the included studies varied significantly in their design, patient characteristics, radiotherapy techniques, toxicity scoring systems, and SNP genotyping methods. This heterogeneity likely contributed to the wide range of reported effect sizes and may reduce the overall comparability and reproducibility of the findings;
2. Risk of bias across studies: the ROBINS-I tool rated many of the studies included in the meta-analysis as having moderate-to-serious risk of bias. Common concerns involved confounding factors, missing outcome data, and inconsistencies in reporting, all of which may have influenced the strength and direction of observed associations;
3. Potential publication bias: Funnel Plot asymmetry and Egger’s regression tests suggested the presence of publication bias, particularly for studies on acute toxicity. This indicates that studies with non-significant or null results may be underrepresented in the literature, potentially leading to overestimating true associations;
4. Limited number of studies and replication: the number of eligible studies—especially those examining late toxicity—remains small. Many gene–toxicity associations were reported in isolated studies and have yet to be replicated, limiting the findings’ generalizability and clinical validity;
5. Lack of standardized protocols: there was no uniform approach to SNP selection, outcome classification, or statistical modeling across studies. This lack of standardization impedes cross-study comparison and hinders the development of robust, reproducible predictive models;
6. Insufficient adjustment for confounders: several studies did not adequately control for important confounding variables such as age, BMI, comorbidities, or specific treatment variables (e.g., radiation dose or technique), which could have influenced toxicity outcomes independently of genetic factors;
7. Variability in toxicity assessment tools: different grading scales (e.g., CTCAE, RTOG, and LENT-SOMA) across studies may have led to inconsistent classification of toxicity severity, complicating pooled analyses and interpretation of effect estimates.

Considering these limitations, future research should focus on well-powered, prospective studies with standardized methodologies and rigorous control for confounding variables. Integrating genomic data into predictive models will require careful validation across diverse cohorts before it can inform clinical decision-making in radiotherapy.

## 6. Conclusions

This meta-analysis confirms previous findings linking genetic markers to the development of acute and late cutaneous toxicities after radiation in breast cancer patients.

Its originality comes in quantifying the major impact of these genetic variants, which reveals that individuals with specific mutations have a 53% greater risk of acute skin toxicities and a 44% higher risk of late toxicities. These findings highlight the possible relevance of genetic profiling in individualized radiotherapy techniques that aim to improve treatment outcomes while minimizing side effects. Nonetheless, the present body of data is hampered by methodological discrepancies, possible biases, and a lack of consistency between studies.

To improve clinical value and dependability, future research should focus standardized assessment techniques, more robust study designs, and broader ethnic and geographical diversity.

## Figures and Tables

**Table 1:** Characteristics of studies on genetic markers and acute radiotherapy-induced skin side effects.

No. Crt.	Author, Year of Publication [Ref.]	Gene (SNP), OR (*p*)	Studied Radiotherapy-Induced Toxicity	Authors’ Conclusions	Forest Plot Study No.
1.	Elisa Eugenia Córdoba et al., 2016 [9]	*GSTP1* (rs1695) 1.08 (*p* = 0.88)	Acute skin toxicity (Grade 2+)	No solid evidence was found for an association between any SNP and acute toxicity.	Acute—Study 1
		*GSTP1* (rs1695) 1.83 (*p* = 0.27)			Acute—Study 2
		*SOD2* (rs4880) 0.63 (*p* = 0.39)			Acute—Study 3
		*SOD2* (rs4880) 0.73 (*p* = 0.57)			Acute—Study 4
		*NOS3* (rs1799983) 0.70 (*p* = 0.45)			Acute—Study 5
		*NOS3* (rs1799983) 1.14 (*p* = 0.82)			Acute—Study 6
		*GSTA1* (rs3957356) 1.71 (*p* = 0.36)			Acute—Study 7
		*GSTA1* (rs3957356) 1.54 (*p* = 0.46)			Acute—Study 8
2.	Delmy Oliva et al., 2018 [10]	*XRCC2* (rs2040639) 6.70 (*p* = 0.007)	Acute radiation skin reactions (ARSR): itching, burning, irritation	XRCC2 rs2040639 SNP associated with burning as ARSR.	Acute—Study 9
		*IFNG* (rs2069705) 3.10 (*p* = 0.01)		IFNG rs2069705 SNP associated with itching as ARSR.	Acute—Study 10
3.	Eunkyung Lee et al., 2020 [11]	*ATM* (rs61915066) 2.64 (*p* = 0.004)	Early adverse skin reactions (EASR), moist desquamation	ATM (rs61915066) associated with RT-induced EASR.	Acute—Study 11
		*CHEK1* (rs11220184) 1.69 (*p* = 0.013)		CHEK1 (rs11220184) associated with RT-induced EASR.	Acute—Study 12
		*ERCC2* (rs60152947) 0.42 (*p* = 0.007)		ERCC2 variants associated with reduced risk of RT-induced EASR.	Acute—Study 13
		*ERCC2* (rs10404465) 0.59 (*p* = 0.007)			Acute—Study 14
		*ERCC2* (rs1799786) 1.74 (*p* = 0.007)			Acute—Study 15
		*TGFB1* (rs4803455) 1.50 (*p* = 0.025)		TGFB1 variants associated with RT-induced EASR.	Acute—Study 16
		*TGFB1* (rs2241714) 0.57 (*p* = 0.04)			Acute—Study 17
		*RAD51C* (rs302877) 0.59 (*p* = 0.006)		RAD51C variants associated with reduced risk of RT-induced EASR.	Acute—Study 18
		*RAD51C* (rs405684) 1.60 (*p* = 0.07)			Acute—Study 19
4.	Harkeran K. Jandu et al., 2023 [12]	GPC5 (rs145328458) 1.23 (*p* = 1.06 × 10^−9^)	Oedema Grade ≥ 2 (2-year follow-up)	A significant association was identified between GPC5 rs145328458 and oedema Grade ≥ 2.	Acute—Study 20
		GPC5 (rs61966612) 1.23 (*p* = 1.23 × 10^−9^)		A significant association was identified for GPC5 rs61966612 with oedema Grade ≥ 2.	Acute—Study 21
		AC093240.1 (rs12657177) 1.24 (*p* = 1.12 × 10^−10^)		A significant association was identified for AC093240.1 rs12657177 with oedema Grade ≥ 2.	Acute—Study 22
		AC093240.1 (rs75912034) 1.24 (*p* = 1.21 × 10^−10^)		A significant association was identified for AC093240.1 rs73151332 with oedema Grade ≥ 2.	Acute—Study 23
5	Soňa Argalácsová et al., 2023 [13]	BRCA1, BRCA2 1.74 (*p* = 0.50)	Acute dermatitis, lymphedema	There is no evidence of increased radiation-induced toxicity for BRCA1/BRCA2 PV carriers.	Acute—Study 24
		CHEK2, ATM, PALB2, RAD51C, RAD51D, BARD1, TP53, FANCM 5.29 (*p* = 0.41)		There is no evidence of increased radiation-induced toxicity for OTHER-PV carriers.	Acute—Study 25
6.	Bahadir Batar et al., 2018 [14]	ERCC1 (rs3212986) 0.21 (*p* < 0.001)	Acute skin reactions	ERCC1 rs3212986 CC genotype may protect radiotherapy-induced acute reactions.	Acute—Study 26
		XPC (rs3731055) 1.00 (*p* = 1.00)		There is no evidence of an association between XPC rs3731055 and radiotherapy-induced acute reactions.	Acute—Study 27
7.	Elisa E. Córdoba et al., 2018 [15]	TNF-α (rs1800629) 5.83 (*p* = 0.22)	Severe radiodermatitis (grades 3 and 4)	There is no evidence of an association between TNF-α (rs1800629) polymorphism and severe radiodermatitis.	Acute—Study 28
		IL-6 (rs1800795) 2.16 (*p* = 0.54)		There is no evidence of an association between IL-6 (rs1800795) polymorphism and severe radiodermatitis.	Acute—Study 29
		ATM (rs1801516) 0.72 (*p* = 0.68)		There is no evidence of an association between ATM (rs1801516) polymorphism and severe radiodermatitis.	Acute—Study 30
8.	Adam J. Webb et al., 2022 [16]	PER3 (rs2087947) 1.27 (*p* = 0.02)	Acute erythema	PER3 rs2087947 genotype influences the occurrence of acute erythema.	Acute—Study 31

**Table 2:** Characteristics of studies on genetic markers and late radiotherapy-induced skin side effects.

No. Crt.	Author, Year of Publication [Ref.]	Gene (SNP), OR (*p*)	Radiotherapy-Induced Toxicity	Authors’ Conclusions	Forest Plot Study No.
1	Cargnin et al., 2021 [17]	TP53 (rs1042522), 1.79 (*p* = 0.028)	Subcutaneous fibrosis and/or telangiectasia (grade 2–3)	No definitive association was established; further studies are required with larger cohorts.	Late—Study 1
		ERCC2 (rs1052555), 1.02 (*p* = 0.922)			Late—Study 2
		ERCC2 (rs13181), 0.90 (*p* = 0.595)			Late—Study 3
		LIG1 (rs7246696), 0.99 (*p* = 0.971)			Late—Study 4
2	Harkeran K. Jandu et al., 2023 [12]	PAX7 (rs643644), 1.40 (*p* = 3.54 × 10^−8^)	Arm lymphoedema G1	PAX7 is associated with an increased risk of arm lymphoedema; replication is recommended.	Late—Study 5
		ATXN7L1 (rs11345494), 1.13 (*p* = 5.78 × 10^−8^)		ATXN7L1 is associated with an increased risk of arm lymphoedema.	Late—Study 6
		ANOS1 (rs188287402), 1.14 (*p* = 2.80 × 10^−8^)	Nipple retraction G2	ANOS1 is associated with an increased risk of nipple retraction.	Late—Study 7
		CRYM/ANKS4B (rs12443861), 1.20 (*p* = 6.17 × 10^−8^)	Telangiectasia G1	CRYM/ANKS4B is associated with an increased risk of telangiectasia.	Late—Study 8
		SLC10A4 (rs34063419), 1.71 (*p* = 1.21 × 10^−8^)	Induration G2	SLC10A4 is associated with an increased risk of induration.	Late—Study 9
		LINC01779 (rs77311050), 1.34 (*p* = 2.54 × 10^−8^)		LINC01779 is associated with an increased risk of induration.	Late—Study 10
3	Aaron J. Grossberg et al., 2018 [18]	TGFB1 (rs1800469), 4.47 (*p* = 0.02)	Subcutaneous fibrosis (grade 2+)	The C−509T allele in TGFB1 is associated with an increased risk of grade 2 or higher fibrosis. Genetic profiling of TGFB1 may help guide treatment decisions.	Late—Study 11
4	Adam J. Webb et al., 2022 [16]	CLOCK (rs1801260), 0.62 (*p* < 0.01)	Late atrophy (2 years post-radiotherapy)	CLOCK (rs1801260), PER3 (rs2087947), and RASD1 (rs11545787) associated with reduced risk of late atrophy (2 years post-radiotherapy).	Late—Study 12
		PER3 (rs2087947), 0.65 (*p* = 0.04)			Late—Study 13
		RASD1 (rs11545787), 0.56 (*p* = 0.02)			Late—Study 14
5	Sebastian Reuther et al., 2015 [19]	ROS Pathway, 1.58 (*p* = 0.045)	Subcutaneous fibrosis	ROS pathway genes have a minor impact on fibrosis but are significant when using a weighted risk model.	Late—Study 15
		DNA Repair, 2.92 (*p* = 0.023)		DNA repair pathway genes are strongly associated with fibrosis using a weighted risk model.	Late—Study 16
		TGFB1 Signaling, 2.68 (*p* = 0.005)		A TGFB1 signaling pathway is strongly associated with fibrosis when using a weighted risk model.	Late—Study 17

**Table 3:** Genes showing significant association with acute skin reactions to radiotherapy.

No. Crt.	Author, Year of Publication [Ref.]	Gene (SNP)—OR (*p*)	Studied Radiotherapy-Induced Toxicity
1.	Delmy Oliva et al., 2018 [10]	*XRCC2* (rs2040639)—6.70 (*p* = 0.007)	Acute radiation skin reactions (ARSRs): itching, burning, irritation.
		*IFNG* (rs2069705)—3.10 (*p* = 0.01)	
2.	Eunkyung Lee et al., 2020 [11]	*ATM* (rs61915066)—2.64 (*p* = 0.004)	Early adverse skin reactions (EASRs), moist desquamation
		*CHEK1* (rs11220184)—1.69 (*p* = 0.013)	
		*ERCC2* (rs60152947)—0.42 (*p* = 0.007)	
		*ERCC2* (rs10404465)—0.59 (*p* = 0.007)	
		*ERCC2* (rs1799786)—1.74 (*p* = 0.007)	
		*TGFB1* (rs4803455)—1.50 (*p* = 0.025)	
		*TGFB1* (rs2241714)—0.57 (*p* = 0.04)	
		*RAD51C* (rs302877)—0.59 (*p* = 0.006)	
		*RAD51C* (rs405684)—1.60 (*p* = 0.07)	
3.	Harkeran K. Jandu et al., 2023 [12]	GPC5 (rs145328458)—1.23 (*p* = 1.06 × 10^−9^)	Oedema Grade ≥ 2 (2-year follow-up)
		GPC5 (rs61966612)—1.23 (*p* = 1.23 × 10^−9^)	
		AC093240.1 (rs12657177)—1.24 (*p* = 1.12 × 10^−10^)	
		AC093240.1 (rs75912034)—1.24 (*p* = 1.21 × 10^−10^)	
4.	Bahadir Batar et al., 2018 [14]	ERCC1 (rs3212986)—0.21 (*p* < 0.001)	Acute skin reactions
5.	Adam J. Webb et al., 2022 [16]	PER3 (rs2087947)—1.27 (*p* = 0.02)	Acute erythema

**Table 4:** Genes showing significant association with late skin reactions to radiotherapy.

No. Crt.	Author, Year of Publication [Ref.]	Gene (SNP) OR (*p*)	Studied Radiotherapy-Induced Toxicity
1.	Cargnin et al., 2021 [17]	TP53 (rs1042522)—1.79 (*p* = 0.028)	Subcutaneous fibrosis and/or telangiectasia (grade 2–3)
2.	Harkeran K. Jandu et al., 2023 [12]	PAX7 (rs643644)—1.40 (*p* = 3.54 × 10^−8^)	Arm lymphoedema G1
		ATXN7L1 (rs11345494)—1.13 (*p* = 5.78 × 10^−8^)	
		ANOS1 (rs188287402)—1.14 (*p* = 2.80 × 10^−8^)	Nipple Retraction G2
		CRYM/ANKS4B (rs12443861)—1.20 (*p* = 6.17 × 10^−8^)	Telangiectasia G1
		SLC10A4 (rs34063419)—1.71 (*p* = 1.21 × 10^−8^)	Induration G2
		LINC01779 (rs77311050)—1.34 (*p* = 2.54 × 10^−8^)	
3.	Aaron J. Grossberg et al., 2018 [18]	TGFB1 (rs1800469)—4.47 (*p* = 0.02)	Subcutaneous fibrosis (grade 2+)
4.	Adam J. Webb et al., 2022 [16]	CLOCK (rs1801260)—0.62 (*p* < 0.01)	Late atrophy (2 years post-radiotherapy)
		PER3 (rs2087947)—0.65 (*p* = 0.04)	
		RASD1 (rs11545787)—0.56 (*p* = 0.02)	

## Appendix A

**Table A1:** Characteristics of studies on genetics markers and acute radiotherapy-induced skin side effects.

Author, Year of Publication [Ref.]	Country/ Period	Study Size— Population	Study Design	Association Gene/Pathway with Radiotoxicity, OR (*p*)	Genetic Testing Methodology	Type of Radiotherapy	Dose and Fractionation	Radiotherapy-Induced Toxicity	Assessment Methods	Statistical Analysis	Authors’ Conclusions	Study No.
Elisa Eugenia Córdoba et al., 2016 [9]	Argentina n/a	80— Breast Cancer Female— Mean age (59 years)	Observational Study	GSTP1 (rs1695) 1.08 (*p* = 0.88)	PCR-based restriction fragment length polymorphism	Conventional RT (50–50.4 Gy, 1.8–2 Gy/fraction) followed by a 12–18 Gy electron boost	50–50.4 Gy, 1.8–2 Gy/fraction; 12–18 Gy electron boost	Acute skin toxicity (Grade ≥ 2)	Radiation Therapy Oncology Group (RTOG) criteria	Univariate logistic regression, Principal Component Analysis (PCA), Odds Ratios (ORs), Confidence Intervals (CIs)	No solid evidence was found for an association between any SNP and acute toxicity.	Acute—Study 1
				GSTP1 (rs1695) 1.83 (*p* = 0.27)								Acute—Study 2
				SOD2 (rs4880) 0.63 (*p* = 0.39)								Acute—Study 3
				SOD2 (rs4880) 0.73 (*p* = 0.57)								Acute—Study 4
				NOS3 (rs1799983) 0.70 (*p* = 0.45)								Acute—Study 5
				NOS3 (rs1799983) 1.14 (*p* = 0.82)								Acute—Study 6
				GSTA1 (rs3957356) 1.71 (*p* = 0.36)								Acute—Study 7
				GSTA1 (rs3957356) 1.54 (*p* = 0.46)								Acute—Study 8
Delmy Oliva et al., 2018 [10]	Sweden February 2011–May 2013	119— Breast Cancer Female— Age > 18	Observational Study	XRCC2 (rs2040639) 6.70 (*p* = 0.007)	Illumina Golden Gate Genotyping assay	Adjuvant RT with two parallel opposing tangential fields using Varian Linacc 2100 CD (3D treatment planning)	50 Gy in 25 fractions; 42.56 Gy in 16 fractions (hypofractionated schedule)	Acute radiation skin reactions (ARSR): itching, burning, irritation	Radiation Therapy Oncology Group (RTOG) scoring system, Visual Analog Scale (VAS)	Odds Ratios (ORs), Confidence Intervals (CIs), Fisher’s exact test, Hochberg method for multiple testing correction	XRCC2 rs2040639 SNP associated with burning as ARSR.	Acute—Study 9
				IFNG (rs2069705) 3.10 (*p* = 0.01)							IFNG rs2069705 SNP associated with itching as ARSR.	Acute—Study 10
Eunkyung Lee et al. 2020 [11]	US December 2008–January 2014	416— Breast Cancer Female— Age > 18	Observational Study	ATM (rs61915066) 2.64 (*p* = 0.004)	Illumina Human Omni2.5–8 v1 genome-wide BeadChip array	Postmastectomy RT, 3D-conformal technique	42.4 to 66 Gy, 3 to 7 weeks	Early adverse skin reactions (EASRs), moist desquamation	Oncology Nursing Society (ONS) scale (0–6)	Multivariable logistic regression, Odds Ratios (ORs), Confidence Intervals (CIs)	ATM rs61915066 is associated with RT-induced EASR.	Acute—Study 11
				CHEK1 (rs11220184) 1.69 (*p* = 0.013)							No evidence of association for CHEK2 with RT-induced EASR.	Acute—Study 12
				ERCC2 (rs60152947) 0.42 (*p* = 0.007)							ERCC2 variants associated with reduced risk of RT-induced EASR.	Acute—Study 13
				ERCC2 (rs10404465) 0.59 (*p* = 0.007)								Acute—Study 14
				ERCC2 (rs1799786) 1.74 (*p* = 0.007)								Acute—Study 15
				TGFB1 (rs4803455) 1.50 (*p* = 0.025)							TGFB1 rs4803455 associated with RT-induced EASR.	Acute—Study 16
				TGFB1 (rs2241714) 0.57 (*p* = 0.04)								Acute—Study 17
				RAD51C (rs302877) 0.59 (*p* = 0.006)							RAD51C variants associated with reduced risk of RT-induced EASR.	Acute—Study 18
				RAD51C (rs405684) 1.60 (*p* = 0.07)								Acute—Study 19
Harkeran K. Jandu et al., 2023 [12]	France, Italy, Belgium, Spain, UK, US 2014–2016	1640— Breast Cancer Female— Mean age (58.5 years)	Case-Control Study	GPC5 (rs145328458) 1.23 (*p* = 1.06 × 10^−9^)	Illumina OncoArrays, genotyping and imputation with SHAPEIT and IMPUTEv2	Whole-breast radiotherapy (adjuvant external beam radiotherapy, 47.9% received IMRT)	50 Gy in a median of 25 fractions (range: 28.5–56.0)	Oedema Grade ≥ 2 (2-year follow-up)	Standard Terminology Criteria for Adverse Events (CTCAE v4.0)	Multivariable generalized linear models, Logistic regression, Odds Ratios (ORs), and Confidence Intervals (CIs).	Significant association identified for GPC5 rs145328458 with oedema Grade ≥ 2.	Acute—Study 20
				GPC5 (rs61966612) 1.23 (*p* = 1.23 × 10^−9^)							Significant association was identified for GPC5 rs61966612 with oedema Grade ≥ 2.	Acute—Study 21
				AC093240.1 (rs12657177) 1.24 (*p* = 1.12 × 10^−10^)							A significant association was identified for AC093240.1 rs12657177 with oedema Grade ≥ 2.	Acute—Study 22
				AC093240.1 (rs75912034) 1.24 (*p* = 1.21 × 10^−10^)							Significant association identified for AC093240.1 rs73151332 with oedema Grade ≥ 2.	Acute—Study 23
Soňa Argalácsová et al., 2023 [13]	Czech Republic March 2015–May 2021	213— Breast Cancer Female— Age >18	Observational Study	BRCA1, BRCA2 1.74 (*p* = 0.50)	Germline next-generation sequencing (CZECANCA multigene panel targeting 226 genes)	Adjuvant radiotherapy (whole-breast irradiation, postmastectomy thorax wall radiation with locoregional lymph nodes)	Various doses, not specified precisely	Acute dermatitis, lymphedema	Common Terminology Criteria for Adverse Events (CTCAE)	Cox proportional hazards model, Gehan–Breslow–Wilcoxon test	No evidence of increased radiation-induced toxicity for BRCA1/BRCA2 PV carriers.	Acute—Study 24
				CHEK2, ATM, PALB2, RAD51C, RAD51D, BARD1, TP53, FANCM 5.29 (*p* = 0.41)							No evidence of increased radiation-induced toxicity for OTHER-PV carriers.	Acute—Study 25
Bahadir Batar et al., 2018 [14]	Turkey n/a	100— Breast Cancer Female— Mean Age (48.7 years)	Observational Study	ERCC1 (rs3212986) 0.21 (*p* < 0.001)	Genotyping by real-time PCR technique	3D conformal radiotherapy	50–66 Gy (2 Gy dose fraction	Acute skin reactions, DNA damage levels, apoptosis	Common Toxicity Criteria (C.T.C.), Micronucleus assay, 8-OHdG assay, TUNEL staining	Pearson’s Chi-square test, Fisher’s exact test, Odds Ratios (ORs), Confidence Intervals (CIs)	ERCC1 rs3212986 CC genotype may provide protection against radiotherapy-induced acute reactions.	Acute—Study 26
				XPC (rs3731055) 1.00 (*p* = 1.00)							There is no evidence of an association between XPC rs3731055 and radiotherapy-induced acute reactions.	Acute—Study 27
Elisa E. Córdoba et al., 2018 [15]	Argentina 2012–2016	125— Breast Cancer Female— Age > 18	Observational Study	TNF-α (rs1800629) 5.83 (*p* = 0.22)	Pyrosequencing	Conventional 3D external beam therapy	Total dose: 50–50.4 Gy, daily fractions of 1.8–2 Gy over 5 weeks	Severe radiodermatitis (grades 3 and 4)	RTOG (Radiation Therapy Oncology Group) scoring system	Chi-square test, Fisher’s exact test, Odds Ratios (ORs), Confidence Intervals (CIs)	There is no evidence of an association between TNF-α G-308A polymorphism and severe radiodermatitis.	Acute—Study 28
				IL-6 (rs1800795) 2.16 (*p* = 0.54)							There is no evidence of an association between IL-6 G-174C polymorphism and severe radiodermatitis.	Acute—Study 29
				ATM (rs1801516) 0.72 (*p* = 0.68)							There is no evidence of an association between ATM G1853A polymorphism and severe radiodermatitis.	Acute—Study 30
Adam J. Webb et al., 2022 [16]	France, Italy, Belgium, Spain, UK, Germany (2014)–2017	1690— Breast Cancer Female— Mean age (58 years)	Case-Control Study	PER3 (rs2087947) 1.27 (*p* = 0.02)	Genotyping using Illumina Infinium OncoArrays	Conventional radiotherapy	Varied across centers, standard fractionation schedules applied	Acute erythema	CTCAE v4.00 scoring system	Logistic Regression, Odds Ratios (ORs), Confidence Intervals (CIs)	PER3 rs2087947 genotype influences the occurrence of acute erythema.	Acute—Study 31

**Table A2:** Characteristics of studies on genetics markers and late radiotherapy-induced skin side effects.

Author, Year of Publication [Ref.]	Country/ Period	Study Size — Population	Study Design	Association Gene/Pathway with Radiotoxicity, OR (*p*)	Genetic Testing Methodology	Type of Radiotherapy	Dose and Fractionation	Radiotherapy-Induced Toxicity	Assessment Methods	Statistical Analysis	Authors’ Conclusions	Study No.
Cargnin et al., 2021 [17]	Italy 1989–2010	285— Breast Cancer Female— Mean age (60.8 years)	Case-Control Study	TP53 (rs1042522), 1.79 (*p* = 0.028)	Targeted NGS and real-time PCR	3D Conformal Radiotherapy	50 Gy in daily fractions of 2 Gy, followed by 9–10 Gy boost for invasive lesions	Subcutaneous fibrosis and/or telangiectasia (grade 2–3)	LENT-SOMA scale (graded annually)	Logistic regression (adjusted for age and BMI), Fisher’s test	No definitive association was established; further studies are required with larger cohorts.	Late—Study 1
				ERCC2 (rs1052555), 1.02 (*p* = 0.922)								Late—Study 2
				ERCC2 (rs13181), 0.90 (*p* = 0.595)								Late—Study 3
				LIG1 (rs7246696), 0.99 (*p* = 0.971)								Late—Study 4
Harkeran K. Jandu et al., 2023 [12]	France, Italy, Belgium, Spain, UK, US 2014–2016	1640— Breast Cancer Female— Mean age (58.5 years)	Case-Control Study	PAX7 (rs643644), 1.40 (*p* = 3.54 × 10^−8^)	Illumina OncoArrays (~600,000 SNPs), Imputation using SHAPEIT and IMPUTEv2	Whole-breast radiotherapy, with or without tumor-bed boost	50 Gy in daily fractions of 2 Gy, with boost for some patients	Arm lymphoedema G1	CTCAE v4.0, Annual follow-up	Logistic regression, Generalized Linear Models, PLINK v2	PAX7 is associated with an increased risk of arm lymphoedema; replication is recommended.	Late—Study 5
				ATXN7L1 (rs11345494), 1.13 (*p* = 5.78 × 10^−8^)				Arm lymphoedema G1			ATXN7L1 is associated with an increased risk of arm lymphoedema.	Late–Study 6
				ANOS1 (rs188287402), 1.14 (*p* = 2.80 × 10^−8^)				Nipple Retraction G2			ANOS1 is associated with an increased risk of nipple retraction.	Late—Study 7
				CRYM/ANKS4B (rs12443861), 1.20 (*p* = 6.17 × 10^−8^)				Telangiectasia G1			CRYM/ANKS4B is associated with an increased risk of telangiectasia.	Late—Study 8
				SLC10A4 (rs34063419), 1.71 (*p* = 1.21 × 10^−8^)				Induration G2			SLC10A4 is associated with an increased risk of induration.	Late—Study 9
				LINC01779 (rs77311050), 1.34 (*p* = 2.54 × 10^−8^)				Induration G2			LINC01779 is associated with an increased risk of induration.	Late—Study 10
Aaron J. Grossberg et al., 2018 [18]	US 2011–2014	287— Breast Cancer Female — Age 40+	Observational Study	TGFB1 (C−509T), 4.47 (*p* = 0.02)	Real-time polymerase chain reaction analyses	Hypofractionated WBI vs. conventionally fractionated WBI	42.56 Gy in 16 fractions or 50 Gy in 25 fractions	Subcutaneous fibrosis (grade 2+)	Physician-assessed fibrosis using LENT/SOMA scale; patient-reported outcomes	Multivariable logistic regression models; Fisher exact test; significance levels reported	The C−509T allele in TGFB1 is associated with an increased risk of grade 2 or higher fibrosis. Genetic profiling of TGFB1 may help guide treatment decisions.	Late—Study 11
Adam J. Webb et al., 2022 [16]	France, Italy, Belgium, Spain, UK, Germany 2014–2017	1690— Breast Cancer Female— Mean age (58 years)	Case-Control Study	CLOCK (rs1801260), 0.62 (*p* < 0.01)	Genotyping using Illumina Infinium OncoArrays (600k SNPs) and imputation using 1000 Genomes Project data	Conventional radiotherapy	Biologically Effective Dose (BED): Acute toxicity = 65 Gy, Late toxicity = 94 Gy	Late atrophy (2 years post-radiotherapy)	CTCAE v4.00, Breast photos	Logistic regression (GLM), mixed effects models, AIC selection	CLOCK (rs1801260), PER3 (rs2087947), and RASD1 (rs11545787) associated with reduced risk of late atrophy (2 years post-radiotherapy).	Late—Study 12
				PER3 (rs2087947), 0.65 (*p* = 0.04)								Late—Study 13
				RASD1 (rs11545787), 0.56 (*p* = 0.02)								Late—Study 14
Sebastian Reuther et al., 2015 [19]	Germany 1988–1995	123 Breast Cancer Female—Age mean (60.1 years)	Observational Study	ROS Pathway, 1.58 (*p* = 0.045)	PCR, TaqMan SNP Genotyping, MassARRAY System	Conventional radiotherapy	Median dose: 55 Gy	Subcutaneous Fibrosis	LENT/SOMA score	Weighted risk model, logistic regression	ROS pathway genes have a minor impact on fibrosis but are significant when using a weighted risk model.	Late—Study 15
				DNA Repair, 2.92 (*p* = 0.023)							DNA repair pathway genes are strongly associated with fibrosis when using a weighted risk model.	Late—Study 16
				TGFB1 Signalling, 2,68 (*p* = 0.005)							Using a weighted risk model, a TGFB1 signaling pathway is strongly associated with fibrosis.	Late—Study 17

**Table A3:** Excluded studies and reason for ineligibility.

No. Crt.	Author, Year of Publication [Ref.]	Reason of Exclusion
1.	Kimi Drobin et al., 2020 [55]	Lack of genetic data: articles lacking information on genetic markers or genetic variations relevant to skin radiotoxicity.
2.	Elahe Abdollahi et al., 2023 [56]	
3.	Elahe Abdollahi et al., 2023 [57]	
4.	Narjes Bakhtari et al., 202 [58]	
5.	Joshua Dulong et al., 2020 [59]	
6.	Yi Cui et al., 2018 [60]	
7.	Leslie A Modlin et al., 202 [61]	
8.	Joanna Huszno et al., 2013 [62]	
9.	Jan Drooger et al., 2015 [63]	
10.	Elnaz Naderi et al., 2023 [64]	
11.	Christoph Weigel et al., 2016 [65]	
12.	Otilia Nuta et al., 2016 [66]	
13.	María J Fuentes-Raspall et al., 2015 [67]	

## Appendix B

**Table A4:** ROBINS-I risk of bias assessment of articles included in the analysis of genetic markers associated with acute radiotherapy-induced skin side effects.

No. Ctr.	Article, Year, [Ref.]	Bias Due to Confounding	Bias in Selection of Participants	Bias in Classification of Interventions	Bias Due to Deviations from Interventions	Bias Due to Missing Data	Bias in Measurement of Outcomes	Bias in Selection of the Reported Result	Overall Bias
1.	Elisa Eugenia Córdoba et al., 2016 [9]	The study controlled for the most important confounding factors, but residual uncontrolled confounding may exist.	Post-intervention variables may have influenced the selection of participants.	Interventions were clearly classified without misclassification or differential misclassification.	Some deviations occurred but were unlikely to affect outcomes significantly.	Some data were missing, but sensitivity analyses suggest minimal impact on results.	Outcome measures were objective and consistently applied across groups.	There was some concern about selective reporting, though most results were transparently reported.	The overall risk of bias is judged as serious due to the serious risk of bias in participant selection and moderate bias in other domains.
2.	Delmy Oliva et al., 2018 [10]	The study does not account for several potential confounders, such as genetic variations unrelated to ARSR.	Participants were consecutively selected from a single hospital. Inclusion and exclusion criteria were clear.	Radiotherapy was uniformly administered based on standard protocols, ensuring consistent classification.	Some participants used corticosteroid cream, while others used moisturizer, leading to differential treatment.	A complete dataset was used for 119 women; minimal missing data were reported.	The Visual Scale is self-reported and may be prone to subjective bias.	Multiple SNPs were tested; only statistically significant results were reported without correction for multiple testing.	The overall risk of bias is assessed as moderate, primarily due to issues related to confounding, outcome measurement, and selection of reported results.
3.	Eunkyung Lee et al., 2019 [11]	Adjusted for significant covariates (race, age, BMI, RT schedule), but potential residual confounding exists.	Patients were part of a well-defined prospective cohort with standardized inclusion criteria.	Radiation therapy was consistently delivered using standardized protocols.	Patients received uniform treatment without deviation from planned interventions.	No missing data were reported for EASRs (Early Adverse Skin Reactions) outcomes, ensuring complete outcome measurement.	Trained physicians performed EASR assessments but could be subjective; the use of a validated scale mitigates this.	The study reports on most outcomes comprehensively but lacks external validation, which may affect reproducibility.	Although the study is well-designed with appropriate adjustments for confounders and comprehensive data collection, the absence of external validation and potential subjectivity in outcome measurement pose a moderate risk of bias.
4.	Harkeran K. Jandu et al., 2023 [12]	The study attempted to control for potential confounders by including clinical and treatment variables in the analysis. However, there may still be unmeasured or residual confounding.	Patients were recruited from multiple centers across Europe and the US. Inclusion and exclusion criteria were clearly defined and applied.	The intervention (radiotherapy) is defined and applied consistently according to protocol at different centers.	Some deviations may have occurred due to different radiotherapy techniques used across centers, but the analysis accounted for these.	Although the study had a relatively large cohort, data points related to certain toxicity endpoints were missing, which could influence the results.	Toxicity assessments were conducted by different clinicians at various sites, potentially introducing measurement variability and bias.	Not all previously reported SNPs associated with toxicity were replicated, and some were excluded from the analysis due to a lack of statistical significance.	The study has a serious risk of bias. The primary weaknesses relate to inconsistencies in outcome measurement and the potential for unmeasured confounding. The findings should be interpreted cautiously, particularly where subjective toxicity measures are involved.
5.	Soňa Argalácsová et al., 2023 [13]	The study attempted to control for important confounders, but residual confounding remains possible.	Interventions were clearly classified according to predefined criteria, with minimal misclassification.	The study had well-defined inclusion criteria and used a clear recruitment process.	Some deviations from the intervention protocol were reported, but they were unlikely to affect the outcome substantially.	Some missing data were reported, but the extent of missingness was insufficient to significantly affect the results.	Measurement of outcomes relied partly on self-reports, which may introduce subjective bias.	Selective reporting was identified, with only statistically significant results being highlighted	The study has a serious risk of bias, due to the presence of serious risk of bias in the selection of reported results, as well as moderate bias in several other domains.
6.	Bahadir Batar et al., 2018 [14]	The study attempts to match cases and controls but does not fully adjust for all potential confounders, such as genetic, lifestyle, and treatment-related factors.	Patients were selected based on well-defined inclusion criteria (newly diagnosed breast cancer patients).	Interventions (radiotherapy) were clearly described and uniformly applied to all participants.	All patients received the same radiotherapy protocol, minimizing bias from deviations.	Data appear complete, with no mention of missing or incomplete datasets.	Measurement of outcomes (acute side effects) was based on self-reports and clinical grading, which may be subjective.	Only specific genes (ERCC1 and XPC) were investigated for polymorphisms. Potential bias may exist due to the selective reporting of results, which focuses on positive findings.	The study generally follows good research practices, but the moderate risk is due to potential confounding factors and selective reporting of results.
7.	Elisa E. Córdoba et al., 2018 [15]	The study considered several confounding variables like age, breast size, and genetic polymorphisms. However, the interactions between these factors were not thoroughly analyzed.	The participants were recruited from a well-defined RT center, and appropriate consent was obtained according to ethical standards.	The study consistently applied standardized radiotherapy protocols (3D external beam therapy) across all participants.	There were no indications of deviations from intended interventions during the study process.	The study mentions the absence of the homozygous minor alleles of certain genes in the population, but it is unclear if the sample size was sufficient to detect these alleles.	Measurement of outcomes (radiodermatitis) was based on clinical observation using the RTOG score. However, subjective assessment may introduce bias.	The reported results match the stated objectives and methodology of the study.	This rating is due to potential bias from confounding variables, possible sample size limitations, and the subjective nature of outcome measurements. However, the study followed standard protocols and properly addressed ethical considerations.
8.	Adam J. Webb et al., 2022 [16]	Potential confounders (e.g., BMI, radiation dose, and surgery type) were controlled for, but differences in grading across sites could introduce bias.	Participants were selected from multiple European sites with different protocols; inclusion criteria were consistent.	Interventions (treatment times) were classified accurately based on solar time; however, not all sites provided consistent timing.	Patients were not randomly assigned to treatment times; treatment time was determined by availability or patient choice.	Missing data were not imputed; incomplete records were omitted, which could bias results.	Outcome measures (e.g., erythema and atrophy) were assessed according to standard protocols but with potential inter-site variability.	Multiple statistical models were tested, and not all were reported; there is a risk of selective reporting.	Based on the domains assessed, this study’s overall risk of bias is rated as “Serious.” The lack of randomization in treatment time assignment and potential variability in outcome measurement across multiple sites are significant sources of bias.

Note: Low risk—Green Color; Moderate risk—Yellow Color; Serious risk—Red Color.

**Table A5:** ROBINS-I risk of bias assessment of articles included in the analysis of genetic markers associated with late radiotherapy-induced skin side effects.

No. Ctr.	Article, Year, [Ref.]	Bias Due to Confounding	Bias in Selection of Participants	Bias in Classification of Interventions	Bias Due to Deviations from Interventions	Bias Due to Missing Data	Bias in Measurement of Outcomes	Bias in Selection of the Reported Result	Overall Bias
1.	Cargnin et al., 2021 [17]	The statistical analysis considered and adjusted for confounders such as age, BMI, and clinical factors, but not all potential confounders may have been accounted for.	All participants were selected based on clear eligibility criteria related to breast cancer diagnosis and treatment. No apparent selection bias was identified.	The intervention (radiotherapy) was applied uniformly to all patients, with no apparent differences in classification.	The study design adhered closely to the planned intervention protocol. No deviations were noted that would impact the results.	Data were collected and reported thoroughly, with no indication of substantial missing data impacting the results.	The outcome assessment relied on clinical observation and patient reports. Though standard scales (LENT-SOMA) were used, there may be some risk of subjective bias.	All results pertinent to the study objectives were reported, with no indication of selective reporting.	Overall, the study has a low risk of bias. The primary concerns relate to the possibility of unmeasured confounders and the subjective nature of outcome measurement.
2.	Harkeran K. Jandu et al., 2023 [12]	The study attempted to control for potential confounders by including clinical and treatment variables in the analysis. However, there may still be unmeasured or residual Confounding.	Patients were recruited from multiple centers across Europe and the US. Inclusion and exclusion criteria were clearly defined and applied.	The intervention (radiotherapy) is defined and applied consistently according to protocol at different centers.	Some deviations may have occurred due to different radiotherapy techniques used across centers, but the analysis accounted for these.	Although the study had a relatively large cohort, data points related to certain toxicity endpoints were missing, which could influence the results.	Toxicity assessments were conducted by different clinicians at various sites, potentially introducing measurement variability and bias.	Not all previously reported SNPs associated with toxicity were replicated, and some were excluded from the analysis due to a lack of statistical significance.	The study has a serious risk of bias. The primary weaknesses relate to inconsistencies in outcome measurement and the potential for unmeasured confounding. The findings should be interpreted with caution, particularly where subjective toxicity measures are involved.
3.	Aaron J. Grossberg et al., 2018 [18]	The study controls for some confounders, but potential unmeasured Confounding could affect the results.	Participants were enrolled according to precise criteria, minimizing selection bias.	Interventions (WBI protocols) are welldocumented and classified appropriately.	No significant deviations from the intended interventions were reported or identified.	There are missing data due to participants lost to follow-up, which could introduce bias.	Outcomes are measured by physicians who are aware of the genotype status, which could introduce bias.	Reported results cover all pre-specified outcomes without evidence of selective reporting.	Overall, the study presents a moderate risk of bias due to potential unmeasured confounding and some missing data.
4.	Adam J. Webb et al., 2022 [16]	Potential confounders (e.g., BMI, radiation dose, surgery type) were controlled for, but differences in grading across sites could introduce bias.	Participants were selected from multiple European sites with different protocols; inclusion criteria were consistent.	Interventions (treatment times) were classified accurately based on solar time; however, not all sites provided consistent timing.	Patients were not randomly assigned to treatment times; treatment time was determined by availability or patient choice.	Missing data were not imputed; incomplete records were omitted, which could bias results.	Outcome measures (e.g., erythema and atrophy) were assessed according to standard protocols but with potential inter-site variability.	Multiple statistical models were tested, and not all were reported; there is a risk of selective reporting.	Based on the domains assessed, the overall risk of bias for this study is rated as “Serious.” The lack of randomization in treatment time assignment and potential variability in outcome measurement across multiple sites are significant sources of bias.
5.	Sebastian Reuther et al., 2015 [19]	The study accounted for known confounders, such as the genes involved in the ROS pathway, DNA repair, and TGFB signalling. However, some potential confounders related to clinical endpoints may not have been adequately adjusted for.	The study included well-defined groups of breast cancer patients who underwent radiotherapy. Ethical approval was obtained, and inclusion criteria were clearly reported.	SNPs were classified based on the prior literature and categorized into pathways, but the approach used to weigh the SNPs could introduce bias, particularly if weighting were not validated externally.	No deviations from intended interventions were reported. The study protocol has been followed consistently.	Some SNPs failed quality control and were excluded from the analysis, which may introduce bias if missingness was not random.	Outcome measurements (RTOG and LENT/SOMA scores for erythema and fibrosis) were well documented, but inter-observer variability and the subjective nature of assessments could introduce some bias.	Multiple statistical models were used without correction for multiple tests. The decision to include or exclude SNPs based on single-SNP analysis could have also introduced selection bias.	While the study had some strengths, including clear endpoints and well-documented methodology, potential bias was introduced by issues related to SNP selection, handling of missing data, and lack of adjustment for multiple testing.

Note: Low risk—Green Color; Moderate risk—Yellow Color; Serious risk—Red Color.
